# A Dual‐Targeting Biomimetic Nanoplatform Integrates SDT/CDT/Gas Therapy to Boost Synergistic Ferroptosis for Orthotopic Hepatocellular Carcinoma Therapy

**DOI:** 10.1002/advs.202413833

**Published:** 2025-01-09

**Authors:** Wen Meng, Ting Chen, Xueping Li, Yi Li, Lu Zhang, Yigang Xu, Tianqiang Song, Ji Qi, Qingqing Xiong, Wen Li

**Affiliations:** ^1^ Tianjin Key Laboratory of Biomedical Materials and Key Laboratory of Biomaterials and Nanotechnology for Cancer Immunotherapy Institute of Biomedical Engineering Chinese Academy of Medical Sciences and Peking Union Medical College Tianjin 300192 China; ^2^ Key Laboratory of Bionic Engineering Ministry of Education Jilin University Changchun 130022 China; ^3^ Department of Hepatobiliary Cancer Liver Cancer Center Tianjin Medical University Cancer Institute & Hospital National Clinical Research Center for Cancer Tianjin Key Laboratory of Digestive Cancer Tianjin's Clinical Research Center for Cancer Tianjin 300060 China; ^4^ State Key Laboratory of Medicinal Chemical Biology Key Laboratory of Bioactive Materials Ministry of Education Frontiers Science Center for Cell Responses and College of Life Sciences Nankai University Tianjin 300071 China

**Keywords:** chemodynamic therapy, gas therapy, hepatocellular carcinoma, nanoenzyme, sonodynamic therapy

## Abstract

The development of efficient therapeutic strategies to promote ferroptotic cell death offers significant potential for hepatocellular carcinoma (HCC) treatment. Herein, this study presents an HCC‐targeted nanoplatform that integrates bimetallic FeMoO_4_ nanoparticles with CO‐releasing molecules, and further camouflaged with SP94 peptide‐modified macrophage membrane for enhanced ferroptosis‐driven multi‐modal therapy of HCC. Leveraging the multi‐enzyme activities of the multivalent metallic elements, the nanoplatform not only decomposes H_2_O_2_ to generate oxygen and alleviate tumor hypoxia but also depletes glutathione to inactivate glutathione peroxides 4, which amplify sonodynamic therapy and ferroptotic tumor death under ultrasound (US) irradiation. Meanwhile, the nanoplatform catalyzes the Fenton reaction to produce hydroxyl radicals for chemodynamic therapy. Elevated intracellular reactive oxygen species trigger the cascade release of CO, leading to lethal lipid peroxidation and further enhancing ferroptosis‐mediated tumor therapy. This nanoplatform demonstrates robust anti‐tumor efficacy under US irradiation with favorable biosafety in both subcutaneous and orthotopic HCC models, representing a promising therapeutic approach for HCC. Additionally, the findings offer new insights into tumor microenvironment modulation to optimize US‐triggered multi‐modal cancer therapy.

## Graphical abstract

This study reports an HCC‐targeted nanoplatform that combines FeMoO_4_ nanoenzyme, CO‐releasing compounds, and SP94‐modified macrophage membrane. This platform improves ferroptosis‐driven multimodal treatment by producing oxygen, depleting glutathione, and creating hydroxyl radicals. It has excellent anti‐tumor activity under ultrasonic irradiation, indicating a feasible technique for treating hepatocellular carcinoma while altering the tumor microenvironment.

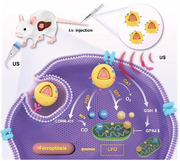

## 1 Introduction

Hepatocellular carcinoma (HCC) ranks as the sixth most commonly diagnosed cancer and the third leading cause of cancer‐related mortality worldwide.^[^ ^1^ ^]^ Due to the insidious nature of HCC, most patients are often diagnosed at advanced stages and are only eligible to systemic treatments.^[^ ^2^ ^]^ Despite the evolution of molecular‐targeted therapy and immunotherapy has dramatically changed the systemic treatment landscape of advanced HCC, the overall response is still limited by the inherent or acquired resistance in cancer cells.^[^ ^3^ ^]^ Meanwhile, systemic therapeutic strategies often cause severe adverse effects.^[^ ^4^ ^]^ Therefore, there is an urgent need to develop a precision therapeutic strategy for more effective treatment of HCC.

Ferroptosis, an iron‐dependent form of non‐apoptotic cell death, is characterized by the excessive accumulation of lethal lipid peroxidation (LPO) resulting from reactive oxygen species (ROS).^[^ ^5^ ^]^ In addition, ferroptosis is also accompanied by glutathione peroxide 4 (GPX4) inactivation mediated by glutathione (GSH) depletion, which further induces redox homeostasis imbalance and tumor cell death.^[^ ^6^ ^]^ Since liver is an important organ for iron storage and metabolism,^[^ ^7^ ^]^ ferroptosis‐based therapy is thus expected as a promising treatment for HCC.^[^ ^8^ ^]^ The occurrence of ferroptosis highly relies on ROS, and it is therefore crucial to develop effective strategies to enhance the intracellular ROS levels for ferroptotic tumor death. To date, various ROS‐generated therapeutic modalities such as chemodynamic (CDT), photodynamic therapy (PDT), radiotherapy and sonodynamic therapy (SDT) have been extensively explored to boost ferroptotic cancer therapy.^[^ ^9^ ^]^ Among these, SDT offers distinct advantages as a non‐invasive treatment modality for deep‐seated tumors such as HCC, owing to the excellent tissue penetration and favorable biosafety profile of ultrasound (US).^[^ ^10^ ^]^

During the process of SDT, sonosensitizers are activated by low‐intensity US to interact with surrounding oxygen to produce highly cytotoxic ROS, and thus results in localized cytotoxicity.^[^ ^10^, ^11^ ^]^ Although a great number of studies have demonstrated the promise of using SDT for tumor therapy, its practical application still faces several challenges.^[^ ^12^ ^]^ First, conventional sonosensitizers suffer from poor solubility, rapid clearance and potential toxicity, which suppresses their SDT efficacy and poses safety issues.^[^ ^13^ ^]^ Second, oxygen consumption caused by rapid tumor growth and insufficient oxygen supply caused by abnormal vasculature led to a hypoxic state of the tumor microenvironment (TME), which greatly restricts the efficacy of oxygen‐dependent SDT.^[^ ^10^, ^14^ ^]^ Meanwhile, high level of reducing GSH in the TME protects cancer cells from oxidative stress, which further impairs SDT‐induced cell death.^[^ ^15^ ^]^ In this regard, it would be of great importance to explore novel sonosensitizers with high efficacy and low toxicity that can modulate the TME to achieve efficient SDT.

In addition, due to the high malignancy and complexity of HCC, SDT monotherapy may sometimes be insufficient to effectively and consistently combat HCC.^[^ ^16^ ^]^ As a result, the development of combined SDT treatment strategies to elevate therapeutic outcomes is urgently expected.^[^ ^17^ ^]^ Gas therapy, which applies gaseous signaling molecules, such as nitric oxide, hydrogen sulfide, and carbon monoxide (CO), has been proposed as a “green” treatment because of its negligible side effects.^[^ ^18^ ^]^ In recent studies, CO has also been demonstrated to drive ferroptosis against cancer through destroying mitochondrial integrity, producing ROS, and downregulating GPX4.^[^ ^18^, ^19^ ^]^ Currently, the localized delivery of external CO to tumor tissues via stimuli‐responsive CO‐releasing molecules (CORMs) represents an advanced category of CO‐based gas therapeutic strategy, greatly reducing the risk of poisoning associated with direct CO inhalation.^[^ ^20^ ^]^ Building on this, the combination of robust SDT with on‐demand CO gas therapy would be a powerful synergistic multi‐modal treatment strategy to induce ferroptosis and achieve effective inhibition against HCC.

**Scheme 1:**
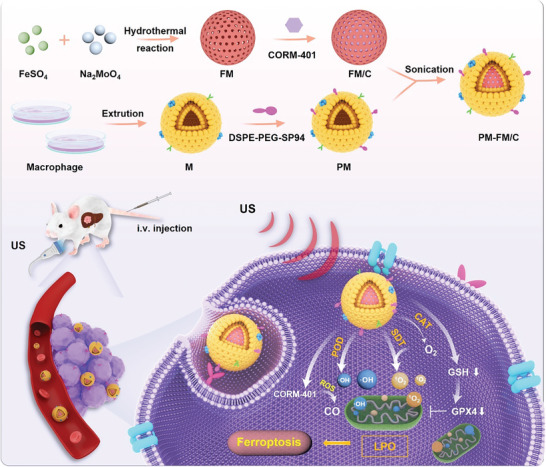
Schematic illustration of the dual‐targeting biomimetic nanoplatform for integrating CDT/SDT/gas therapy to boost synergistic ferroptosis against orthotopic HCC. The CO‐releasing molecule CORM‐401 was encapsulated into bimetallic compound FM to form a muti‐modal therapeutic nanoplatform (FM/C), which was further cloaked with macrophage membrane inserted with SP94‐peptide to endow dual‐targeting ability toward HCC. The multi‐enzyme activity of FM not only promoted CDT to generate ·OH but also improved SDT performance to produce ^1^O_2_ by alleviating hypoxia, which in turn activated CO release. As a result, the depletion of GSH and high level of ROS led to the accumulation of lethal lipid peroxidation (LPO) to promote ferroptosis‐based tumor death in an orthotopic HCC model.

Inorganic nanomaterials, featuring superior physicochemical properties and versatility, have recently been explored as a new generation of sonosensitizers.^[^ ^21^ ^]^ By exploiting multivalent elements (Fe^2+^/^3+^, Cu^+^/Cu^2+^, Mo^4+^/Mo^6+^, Co^2+^/^3+^, etc.), some inorganic nanomaterials can mimic natural enzymes and exhibit high catalytic activity in the TME.^[^ ^22^ ^]^ Herein, we explored a bimetallic compound, FeMoO_4_ nanoparticles (FM), as a sonosensitizer and investigated its role in modulating the TME to achieve enhanced SDT upon US irradiation. Thereafter, CORM‐401, a commercially available oxidant‐sensitive CORM (Figure S1, Supporting Information),^[^ ^23^ ^]^ was readily loaded into FM nanoparticles to form FM/C nanoparticles for synergistic multi‐modal treatment of HCC. In order to achieve the specific accumulation of therapeutic agents at the HCC tumor, macrophage membrane inserted with SP94‐peptide was further cloaked on FM/C to construct a dual‐targeting biomimetic nanoplatform (PM‐FM/C) for subcutaneous and orthotopic HCC therapy (**Scheme** **1**). Leveraging the multi‐enzyme activities, FM could render Fenton reaction to produce hydroxyl radical (·OH) for CDT and catalyze the decomposition of H_2_O_2_ to produce O_2_ to relieve hypoxia, which improved its SDT performance even in a hypoxic environment. The high level of intracellular ROS induced the cascade release of CO, which together led to the accumulation of lethal LPO for inducing ferroptotic cancer cell death. Meanwhile, the nanoplatform can react with reducing species in the TME for GSH depletion, which further elevated the levels of LPO through the inactivation of GPX4. Our findings show that PM‐FM/C induces robust ferroptosis‐based tumor death under US irradiation and holds great potential to serve as an efficient multi‐modal therapeutic agent for HCC therapy.

## 2 Results and Discussion

### 2.1 Preparation and Characterization of FM

**Figure 1:**
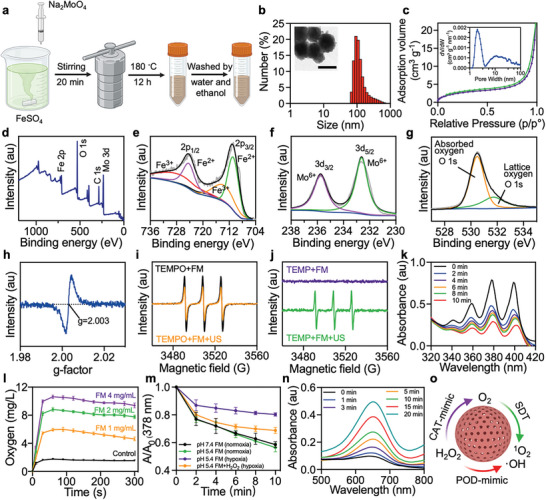
Synthesis and characterization of FM. a) The illustration of the synthesis of FM. The figure was created in BioRender.com. b) Representative DLS result and TEM image (inserted) of FM. Scale bar: 100 nm. c) N_2_ absorption–desorption curves and pore size distribution curve (inserted) of FM. d) XPS survey scan spectrum of FM. e) Fe 2p, f) Mo 3d, and g) O 1s peaks in the XPS spectra of FM. h) Oxygen vacancy detected by ESR of FM. i) ESR spectra of electron holes trapped by TEMPO in the presence of FM upon US irradiation. j) ESR spectra of ^1^O_2_ trapped by TEMP in the presence of FM upon US irradiation. k) The ^1^O_2_ generation ability of FM upon US irradiation detected by ABDA. l) The O_2_ release profiles of FM at different concentrations in the presence of H_2_O_2_. m) Plots of A/A_0_ of ADBA in the presence of FM versus US irradiation time under different conditions, where A_0_ and A are the absorbance of ABDA (378 nm) before and after US irradiation, respectively. n) The ^•^OH generation ability detected by the TMB probe in the presence of H_2_O_2_. o) Schematic illustration delineating the multi‐enzyme catalytic ability of FM for enhanced ROS generation. Data are presented as mean ± SD (*n* = 3).

FM was first synthesized by the hydrothermal reaction of Na_2_MoO_4_·2H_2_O and FeSO_4_·7H_2_O (**Figure** **1**a). The hydrothermal synthesis method has been widely applied to produce various classes of inorganic materials with porous nanostructure.^[^ ^24^ ^]^ Dynamic laser scattering (DLS) analysis revealed that the as‐synthesized FM had an average size of 106 nm, and the inserted transmission electron microscopy (TEM) image showed a uniform spherical morphology with the rough edge (Figure 1b). The zeta potential of FM was measured to be 36.41 mV (Figure S2, Supporting Information). The FTIR spectrum displayed a sharp band at 916 cm^−1^ with a shoulder at 960 cm^−1^, which was attributed to the stretching and bending vibrations of Mo = O and Mo‐O‐Mo, respectively (Figure S3, Supporting Information). Moreover, the absorption band at 624 cm^−1^ could be ascribed to the stretching vibration of Mo‐O‐Fe. The N_2_ adsorption‐desorption analysis clearly showed that FM has type II isothermal curves (Figure 1c), indicating the microporous structure of FM. Furthermore, two peaks were observed in the pore size distribution ranging from 1 to 100 nm (Figure 1c), suggesting that some mesopores coexist with the macropores. The average pore diameter was calculated to be 13.8 nm. During the hydrothermal process, high hydrothermal temperature promotes extensive nucleation of primary particles from homogeneous aqueous solution at the early stages, followed by the aggregation of these primary particles into spherical structures with voids between them.^[^ ^25^ ^]^ Therefore, we deduced that micro‐ and mesoporous structures of FM were achieved as a result of aggregate recrystallization.

X‐ray photoelectron spectroscopy (XPS) measurements were performed to characterize the surface elemental compositions of FM (Figure 1d). The survey scan displayed the typical peaks of Fe, Mo, and O elements. The high‐resolution XPS spectrum of Fe 2p revealed the coexistence of Fe^2+^ oxidation state (710.77 and 724.27 eV) with some Fe^3+^ species (713.64 and 727.71 eV) (Figure 1e), while the Mo 3d spectrum demonstrated the presence of Mo^6+^ oxidation state (232.6 and 235.8 eV) within FM (Figure 1f). Moreover, the asymmetric peak in the O 1s spectrum could be deconvoluted into two peaks associated with lattice oxide (530.4 eV) and absorbed oxygen (531.6 eV), respectively (Figure 1g). The electron spin resonance (ESR) spectrum showed a characteristic signal peak with g‐factor of 2.003, further confirming the oxygen‐deficient structure of FM (Figure 1h). The 4‐amino‐2,2,6,6‐tetramethylpiperidine‐1‐oxyl (TEMPO) probe analysis depicted in Figure 1i confirmed the presence of electron holes within FM after US irradiation, which is advantageous for sonodynamic performance. Moreover, singlet oxygen (^1^O_2_) signals trapped by 2,2,6,6‐tetramethylpiperidine (TEMP) were evidently displayed in the ESR spectrum of FM after US stimulation for 10 min (Figure 1j). The above results confirmed the successful synthesis of bimetallic FeMoO_4_ nanoparticles, and their abundant oxygen vacancies as well as the US‐induced separation of electron‐hole pairs contributed to the effective sonosensitizing properties of FM.

### 2.2 Multi‐Enzyme Catalytic Ability of FM for Enhanced ROS Generation

To evaluate the sonodynamic performance of FM, 9,10‐anthracenediyl‐bis(methylene)dimalonic acid (ABDA) was used to detect ^1^O_2_ generation under US irradiation. As displayed in Figure 1k, the characteristic peaks of ADBA progressively decreased with the increase of US irradiation time, indicating the sustained generation of ^1^O_2_. Considering the hypoxic microenvironment at the tumor site, we also performed ^1^O_2_ detection in deoxygenated FM solution upon US irradiation, and it was undoubtedly that ^1^O_2_ production efficiency was remarkably inhibited under hypoxic condition (Figure S4a, Supporting Information). In this regard, we further examined the multi‐enzyme catalytic activities of FM and the regulatory roles in the TME for enhancing ROS generation.

In view of the high concentration of H_2_O_2_ at the tumor site, the catalase (CAT)‐mimicking activity to decompose H_2_O_2_ to produce O_2_ was first evaluated by detecting the dissolved oxygen concentration in the solutions containing FM (1, 2, or 4 mg mL^−1^) with the addition of H_2_O_2_. Dissolved oxygen increased sharply upon H_2_O_2_ addition, and O_2_ production increased with FM concentration up to 4 mg mL^−1^ (Figure 1l). During the incubation of 5 min, dissolved oxygen was maintained at a constant level with only a slight decrease. As expected, the ^1^O_2_ generation ability was partially restored by the addition of H_2_O_2_, as the value of A/A_0_ (378 nm) of the “FM+H_2_O_2_ (hypoxia)” group was higher than that of the “FM (hypoxia)” group (Figures 1m and S4b, Supporting Information). The elevated ^1^O_2_ production efficiency in the presence of H_2_O_2_ was attributed to the CAT‐like catalytic effect of FM to generate O_2_, which subsequently provided a local reoxygenated atmosphere to promote US‐triggered ^1^O_2_ generation. Therefore, FM itself could not only serve as an excellent acoustic sensitizer, but also ameliorate hypoxia by utilizing its CAT‐mimicking activity for oxygen production, thereby promoting efficient ROS under ultrasound stimulation.

The existence of Fe^3+^/Fe^2+^ in FM motivated us to evaluate the peroxidase (POD)‐like activity of FM by the oxidation of 3,3,5,5‐tetramethylbenzidine (TMB) upon H_2_O_2_ addition. The absorbance peak at 652 nm of the oxidant TMB gradually increased upon H_2_O_2_ addition in FM solution at pH 5.4, indicating the dramatic generation of ·OH (Figure 1n). In contrast, the oxidation of TMB was weak at pH 7.4 (Figure S5, Supporting Information). The Fe^3+^/Fe^2+^ couple endowed FM with POD‐like activity by rendering a Fenton reaction with H_2_O_2_ to produce ·OH under acidic condition. Glutathione (GSH), as an important intracellular metabolite, can scavenge the ·OH and increase the resistance of cancer cells to oxidative stress. As shown in Figure S6 (Supporting Information), FM could effectively consume GSH, and the conversion efficiency of GSH exhibited a significant concentration‐dependent elevation. Overall, the multi‐enzyme catalytic activities of FM allowed it to consume excess H_2_O_2_ in the TME not only to improve the US‐triggered ^1^O_2_ generation by ameliorating hypoxia but also to generate ·OH, resulting in a local ROS burst to produce SDT/CDT effects (Figure 1o).

### 2.3 Preparation and Characterization of Dual‐Targeting Biomimetic PM‐FM/C

**Figure 2:**
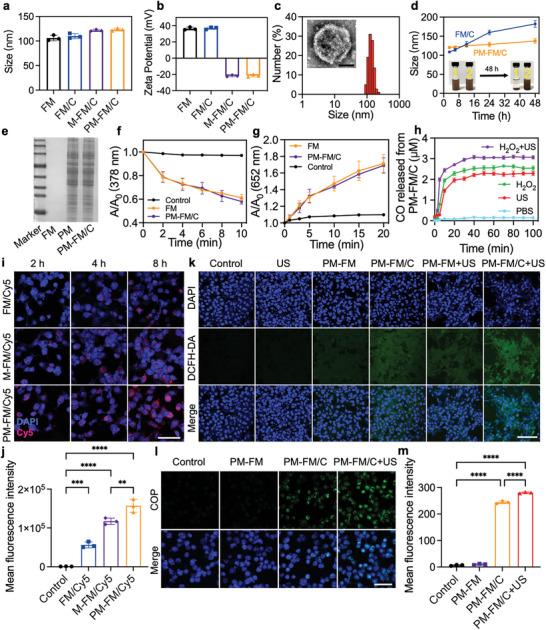
Characterization of PM‐FM/C. a) The average sizes and b) zeta potentials of FM, FM/C, M‐FM/C and PM‐FM/C. c) Representative DLS result and TEM image (inserted) of PM‐FM/C. Scale bar: 100 nm. d) Average size change of FM/C and PM‐FM/C versus the storage time and the inserted photo of FM/C and PM‐FM/C dispersed in PBS for 48 h (1: FM/C, 2: PM‐FM/C). e) Protein analysis of FM, SP94 modified macrophage membrane (PM), and PM‐FM/C by SDS‐PAGE. f) Plots of A/A_0_ of TMB in the presence of FM or PM‐FM/C upon H_2_O_2_ addition for different time points, where A_0_ and A are the absorbances of TMB (652 nm) before and after H_2_O_2_ addition, respectively. g) Plots of A/A_0_ of ADBA in the presence of FM or PM‐FM/C versus US irradiation time, where A_0_ and A are the absorbance of ABDA (378 nm) before and after US irradiation, respectively. h) Profiles of CO release from PM‐FM/C under different conditions. i) Representative CLSM images of Hepa1‐6 cells after incubation with FM/Cy5, M‐FM/Cy5 and PM‐FM/Cy5 for different time. Scale bar: 100 µm. j) The mean fluorescence intensity of Cy5 in Hepa1‐6 cells after different treatments for 4 h was detected by flow cytometry. k) Representative CLSM images of cellular ROS generation in Hepa1‐6 cells using DCFH‐DA as a probe. Scale bar: 100 µm. l) Representative CLSM images of Hepa1‐6 cells after different treatments and m) The corresponding mean fluorescence intensity of cellular CO release using COP as a probe. Scale bar: 100 µm. Data are presented as mean ± SD (*n* = 3). ^**^ *p* < 0.01, ^***^ *p* < 0.001, ^****^ *p* < 0.0001, and statistical analysis was performed using one‐way ANOVA.

Due to the microporous structure, FM is exploited to accommodate CORM‐401 to obtain FM/C. Inductively coupled plasma‐Mass‐spectrometry (ICP‐MS) analysis showed that the encapsulation efficiency of CORM‐401 in FM/C reached 58.03% when the incubation molar ratio of FM/CORM‐401 was 1/1. The average size and zeta potential of FM/C were measured as 110 nm and 35.32 mV (**Figure** **2**a,b), which had no obvious difference with the pristine FM, indicating that CORM‐401 loading had a negligible influence on FM. In addition, the TEM image of FM/C was similar to that of FM (Figure S7, Supporting Information).

To improve the stability of FM/C and confer HCC targeting ability, the Raw264.7 cell membrane was extracted and modified with SP94‐conjugated DSPE‐PEG_2K_ (DSPE‐PEG_2K_‐SP94) via the insertion of hydrophobic DSPE into the membrane bilayer, and then cloaked onto FM/C by sonication to yield the dual‐targeting biomimetic PM‐FM/C (Scheme 1). After SP94‐modified cell membrane coating, the average hydrodynamic diameter of PM‐FM/C increased to 123 nm (Figure 2a,c) and the zeta potential became negative at −21.54 mV (Figure 2b). The TEM image showed that a thin layer of cell membrane was observed on the surface of PM‐FM/C (Figure 2c). As expected, the stability and dispersity of PM‐FM/C were apparently improved compared to FM/C, as aggregation and precipitation were not observed after 48 h in PBS (Figure 2d). In addition, PM‐FM/C showed protein bands similar to SP94‐modified macrophage membrane (PM) (Figure 2e), which further suggested the cell membrane was successfully camouflaged on the surface of PM‐FM/C.

### 2.4 ROS Generation and CO Release of PM‐FM/C In Vitro

Following the fabrication of PM‐FM/C, we validated the ROS generation abilities upon US stimulation or H_2_O_2_ addition. As revealed in Figure S8 (Supporting Information), PM‐FM/C retained the ability to produce ^1^O_2_ as a sonosensitizer as well as the POD‐like activity for ·OH generation. Compared to unencapsulated FM, the ROS generation abilities of PM‐FM/C exhibited negligible changes which suggested that PM modification did not significantly affect the CDT and SDT performance of FM (Figure 2f,g).

After confirming the ROS generation abilities of PM‐FM/C, we then monitored the ROS‐activated CO release based on the high binding affinity between hemoglobin (Hb) and CO. The adsorption peak at 410 nm attributed to HbCO gradually increased, while that at 430 nm associated with Hb decreased in the UV spectra of Hb, indicating the sustained dissociation of CO with the prolonged time (Figure S9, Supporting Information). The release of CO from PM‐FM/C with different treatments was compared and the result was displayed in Figure 2h. The CO release was negligible without any treatments. In contrast, the concentration of dissolved CO was rapidly increased upon H_2_O_2_ addition or increased with prolonged US irradiation time. Furthermore, PM‐FM/C showed the highest CO‐released efficiency when it was simultaneously exposed to US irradiation and in the presence of H_2_O_2_. The above results indicated that ·OH produced by the Fenton reaction and ^1^O_2_ generated by US activation synergistically promoted the release of CO from PM‐FM/C.

### 2.5 ROS Generation and CO Release of PM‐FM/C at Cellular Level

Considering the dual‐targeting nature of SP94‐modified macrophage membrane against HCC, the cellular uptake of the biomimetic nanosystem toward Hepa1‐6 cells was first investigated by flow cytometry and confocal laser scanning microscopy (CLSM). Fluorescent dye Cy5 was chosen as a replacement for CORM‐401 and loaded into FM to construct naked FM/Cy5, macrophage membrane cloaking M‐FM/Cy5 and SP94‐modified macrophage membrane cloaking PM‐FM/Cy5 for Hepa1‐6 cells incubation. The CLSM images of Hpea1‐6 cells showed that the cellular uptake of PM‐FM/Cy5 increased over the prolonged time, and the highest Cy5 fluorescence was detected in the cells treated with PM‐FM/Cy5, as compared to the other two groups (Figure 2i). Comparison of the flow histogram profiles of Hepa1‐6 cells at 4 h exhibited the same trend (Figure S10, Supporting Information). Quantitative analysis by flow cytometry indicated that the Cy5 fluorescence intensity in PM‐FM/Cy5‐treated cells was significantly higher than that in PM/Cy5‐ and FM/Cy5‐treated cells, by 1.34‐fold and 2.80‐fold, respectively (Figure 2j). The above results demonstrated the superior targeting capability of PM‐FM/Cy5 to HCC cells, which was facilitated by the macrophage membrane and SP94 peptide coating.

Subsequently, we examined the ROS generation of PM‐FM/C in Hepa1‐6 cells by visualizing the intracellular ROS levels using DCFH‐DA as a probe. Negligible green fluorescence signal was observed in the control and US‐only groups. Slight green fluorescence signal was detected in the PM‐FM and PM‐FM/C groups, indicating the minor ROS production generated by the Fenton‐like reaction with the endogenous H_2_O_2_ in Hepa1‐6 cells (Figure 2k). Besides, stronger green fluorescence was detected in the PM‐FM/C group, indicating that CO release also promoted ROS generation.^[^ ^26^ ^]^ In contrast, US irradiation significantly elevated the intracellular ROS level, as stronger fluorescence signal was detected in the PM‐FM+US and PM‐FM/C+US groups (Figure 2k). Flow cytometric analysis of DCFH‐DA fluorescence in Hepa1‐6 cells and the statistical analysis of mean fluorescence intensity further confirmed the results (Figure S11, Supporting Information). The ROS‐activated CO release property of PM‐FM/C was further evaluated in Hepa1‐6 cells using COP (a CO fluorescent probe). As depicted in Figure 2l,m, green fluorescence was observed in the cells treated with PM‐FM/C, revealing the CO release triggered by the endogenous H_2_O_2_. In contrast, stronger green fluorescence was obviously observed in PM‐FM/C+US group, indicating the large amount of CO release due to the elevated ROS level under US irradiation. Given all the above findings, it can be concluded that US‐triggered PM‐FM/C not only generated ROS for rendering CDT/SDT effect in the tumor environment but also mediated cascade‐activated CO release for gas therapy, which may result in synergistic therapeutic effects for cancer.

### 2.6 In Vitro Synergistic Therapeutic Effect of PM‐FM/C

**Figure 3:**
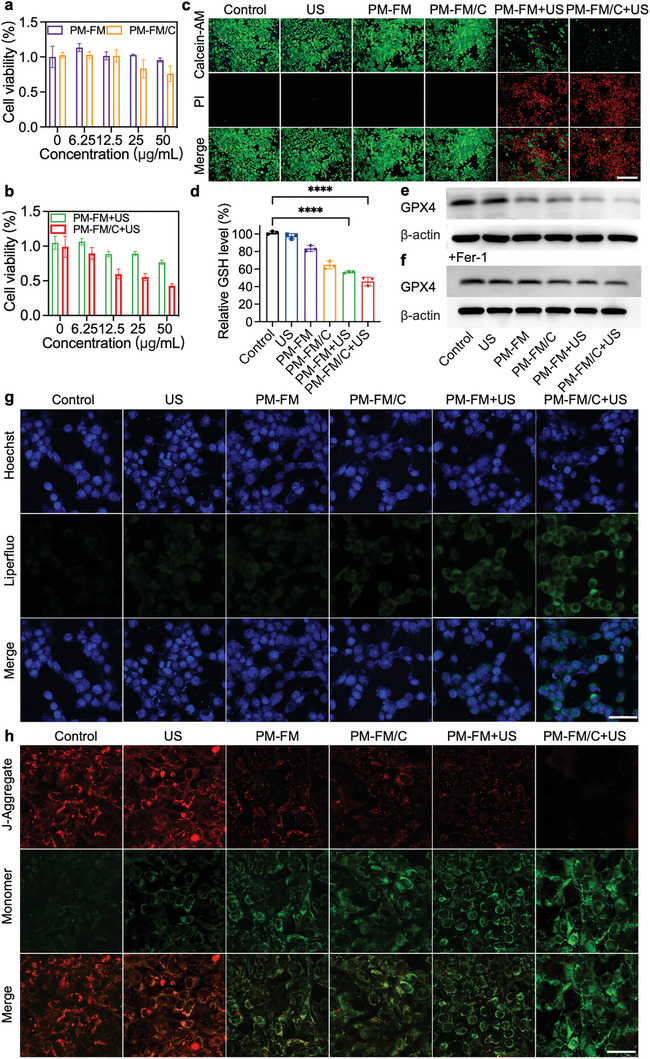
In vitro anti‐tumor effects of PM‐FM/C. Cell viabilities of Hepa1‐6 cells treated with PM‐FM or PM‐FM/C without a) or with b) US irradiation. c) Representative fluorescence images of Hepa1‐6 cells stained with calcein‐AM and PI after different treatments as indicated. Scale bar: 100 µm. d) Relative GSH level detected in Hepa1‐6 cells after different treatments as indicated. e) Western blot analysis of GPX4 expression in Hepa1‐6 cells after different treatments. f) Western blot analysis of GPX4 expression in Hepa1‐6 cells pretreated with Fer‐1 prior to various treatments. g) Representative CLSM images of lipid peroxidation on Hepa1‐6 cells membrane with different treatments as indicated using Liperfluo as a probe. Scale bar: 50 µm. h) Representative CLSM images of Hepa1‐6 cells stained with JC‐1 after different treatments as indicated. Scale bar: 50 µm. Data are presented as mean ± SD (*n* = 3). ^****^ *p* < 0.0001 using one‐way ANOVA.

Given that PM‐FM/C could target Hepa1‐6 cells to facilitate cell internalization and then generate significant amounts of ROS and CO under US stimulation, we further explored the in vitro therapeutic potential against Hepa1‐6 cells. The biosafety and compatibility of PM‐FM and PM‐FM/C were first evaluated in normal 293T cells by CCK‐8 assay. The cell viabilities of 293T cells remained above 95.0% after 24 h incubation with PM‐FM or PM‐FM/C at the concentrations ranging from 6.25 to 50 µg mL^−1^ (Figure S12, Supporting Information), indicating that PM‐FM and PM‐FM/C had negligible cytotoxicity to normal cells. In comparison, concentration‐dependent cytotoxicity was observed in Hepa1‐6 cells (**Figure** **3**a), which was perhaps due to the intrinsic H_2_O_2_ in tumor cells. Notably, Hepa1‐6 cell viability significantly decreased following US irradiation (Figure 3b), with the PM‐FM and PM‐FM/C groups exhibiting viabilities of 76.7% and 42.9%, respectively, at a concentration of 50 µg mL^−1^. The therapeutic efficacy was further confirmed by calcein‐AM and propidium iodide (PI) double‐staining assay. As depicted in Figure 3c, the PM‐FM/C+US group exhibited the highest proportion of dead cells as indicated by the strongest red fluorescence signal. The enhanced cytotoxicity of PM‐FM/C under US irradiation further emphasized the critical role of elevated ROS and CO levels in tumor cell killing, stemming from the multi‐catalytic reactions, US‐triggered sonodynamic effect, and cascade‐activated CO release.

Ferroptosis is a regulatory cell death associated with excessive LPO that ultimately results in membrane rupture. We then investigated the ferroptosis‐related indicators in Hepa1‐6 cells after various treatments. Reductive GSH serves as the co‐substrate of GPX4 to balance the oxidative level in cells. Generally, LPO accumulation process involves the depletion of the intracellular GSH to promote GPX4 inactivation.^[^ ^6b^ ^]^ As except, the intracellular GSH content was decreased to 43.19% in the cells treated with PM‐FM/C+US group as compared to that in the cells without any treatments (Figure 3d). Besides, PM‐FM and PM‐FM/C significantly decreased GPX4 expression, and the introduce of US irradiation after culture with PM‐FM and PM‐FM/C further promoted GPX4 inhibition (Figures 3e and S13a, Supporting Information). Ferrostatin‐1 (Fer‐1), an inhibitor of ferroptosis, was used for the recovery experiment, and the results showed that the decrease of GPX4 expression was dramatically alleviated (Figures 3f and S13b, Supporting Information), which further confirmed the involvement of GPX4 regulation in the induction of ferroptosis by PM‐FM/C upon US treatment in Hepa1‐6 cells. Furthermore, lipid peroxidation induced by different treatments was detected in Hepa1‐6 cells. As shown in Figure 3g, relatively stronger green fluorescence was found in the PM‐FM+US and PM‐FM/C+US groups in comparison with the other groups. C11‐BODIPY581/591, an additional LPO‐specific fluorescent probe, was further used to indicate LPO, and the same trend was observed (Figures S14 and S15, Supporting Information). Noticeably, the lipid peroxidation of the PM‐FM/C group in Figure 3g was not as obvious as the ROS generation as shown in Figure 2k. This may be attributed to the following reasons. First, although ROS was generated by PM‐FM/C treatment, lipid peroxidation usually required a longer duration of ROS exposure or higher sustained ROS levels to accumulate detectable damage, which may show a more delayed response. Moreover, the conditions (e.g., cell culture time and assay sensitivity) of these two individual experiments may also differ, leading to different outcomes in ROS detection and lipid peroxidation.

During the process of ferroptosis, mitochondrial damage is considered to be one of the key factors leading to cell death. We then evaluated mitochondrial function by detecting the mitochondrial membrane potentials (MMPs) using 5,5′,6,6′‐tetrachloro‐1,1′,3,3′‐tetraethyl‐imidacarbocyanine iodide (JC‐1) as a probe. In general, JC‐1 aggregates in the mitochondrial matrix can produce red fluorescence when staining healthy mitochondria with higher potential. However, JC‐1 monomers cannot aggregate in the mitochondrial matrix and produce green fluorescence when staining damaged mitochondria with lower potential. Compared to the control and US alone groups, the PM‐FM/C+US group induced a significant increase in green JC‐1 monomers (Figure 3h), indicating the damage of the mitochondrial membrane.

### 2.7 Therapeutic Mechanisms of PM‐FM/C at Transcriptome Levels

**Figure 4:**
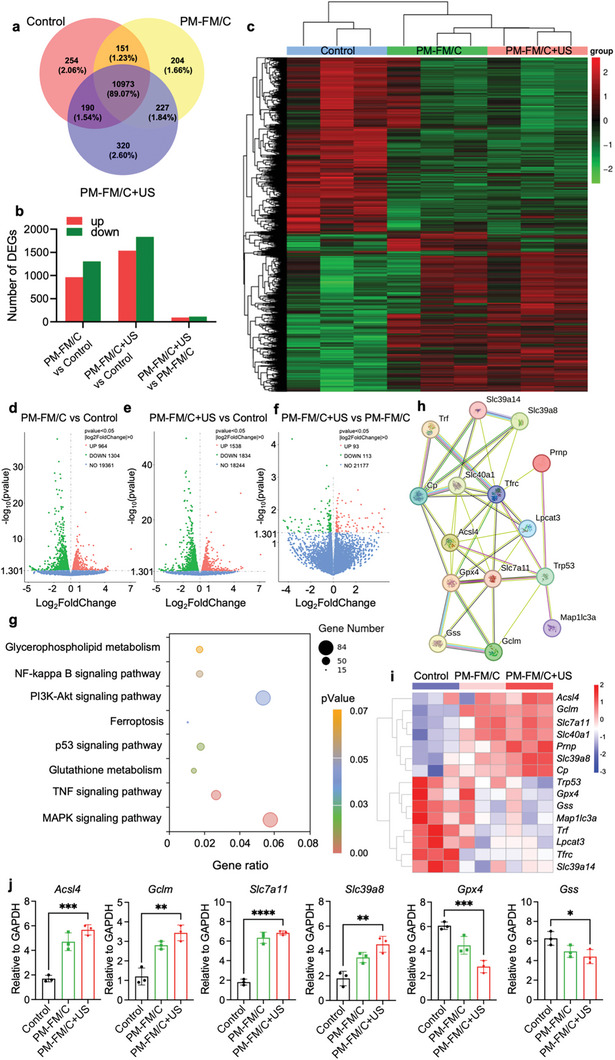
Transcriptional analysis of Hepa1‐6 cells after different treatments. a) VENN graph revealed the number of co‐expressed genes. b) Statistic of up‐and down‐regulated genes in different comparisons. c) The heat map of DEGs in Hepa1‐6 cells with different treatments as displayed. Volcano plots of differentially expressed genes in d) PM‐FM/C versus control, e) PM‐FM/C+US versus control, and f) PM‐FM/C+US versus PM‐FM/C. g) KEGG pathway enrichment analysis. h) Protein–protein interaction networks and the significant gene module in the ferroptosis‐related gene set. i) Heatmap of ferroptosis‐related DEGs identified by RNA‐seq in Hepa1‐6 cells with different treatments as indicated. j) Validation of the mRNA level of representative ferroptosis‐related genes by RT‐PCR. Data are presented as mean ± SD (*n* = 3). ^*^ *p* < 0.05, ^**^ *p* < 0.01, ^***^ *p* < 0.001, ^****^ *p* < 0.0001, and statistical analysis was performed using one‐way ANOVA.

To further elucidate the therapeutic mechanisms of US‐triggered PM‐FM/C at the molecular level, the transcriptome analysis was performed to analyze the mRNA expression patterns of Hepa1‐6 cells after different treatments. Approximately 11100 genes were co‐expressed in the three groups (**Figure** **4**a). Pearson correlation between different samples demonstrated the reality of the RNA‐sequencing data (Figure S16, Supporting Information). A total of 24855 genes were identified through gene expression analysis. Meanwhile, thousands of differentially expressed genes (DEGs) were detected in the samples with different treatments (Figure 4a,b). The heat map further revealed an evident transcriptomics discrepancy in gene expression among the three groups (Figure 4c). Compared to the control group, PM‐FM/C group exhibited 2268 (964 up‐regulated and 1304 down‐regulated, Figure 4d), while the PM‐FM/C+US group exhibited 3372 DEGs (1538 up‐regulated and 1834 down‐regulated, Figure 4e). In addition, 206 DEGs were detected in PM‐FM/C+US versus PM‐FM/C, including 93 up‐regulated genes and 113 down‐regulated genes (Figure 4f). The results indicated that PM‐FM/C treatment exerted a more obvious effect on the change of gene expression pattern after US stimulation.

Based on the DEGs between PM‐FM/C+US and the control group, gene ontology (GO) and Kyoto Encyclopedia of Genes and Genomes (KEGG) enrichment analysis were further performed to unveil the biological function and pathways associated with the altered expression genes. GO results revealed that the phospholipid biosynthesis process was most affected in the cells treated with PM‐FM/C plus US stimulation versus the control cells (Figure S17, Supporting Information). KEGG analysis indicated that the ferroptosis pathway, along with related signaling pathways such as MAPK, TNF, and p53, were associated with the therapeutic mechanisms of PM‐FM/C+US (Figure 4g). Gene set enrichment analysis (GSEA) also demonstrated that the treatment with PM‐FM/C+US irradiation changed the expression of genes associated with ferroptosis (Figure S18, Supporting Information). Inspired by the above analysis as well as the ferroptosis‐inducing effect verified at the cellular level, we further performed psychophysiological interaction (PPI) analysis (Figure 4h) and generated a heat map diagram (Figure 4i) on the 15 differentially expressed ferroptosis‐related genes (FRGs) enriched by KEGG analysis.

The heat map depicted that 7 FRGs were up‐regulated and 8 FRGs were down‐regulated in PM‐FM/C‐treated cells, while the changes in gene expression were further enhanced by US irradiation. These genes were mainly associated with cystine/glutamate transport (*Slc7a11*), iron transport (*Trf*, *Tfrc*, *Cp*), glutathione metabolism (*Gclm*, *Gss*, *Gpx4*), and arachidonate metabolism (*Acsl4*), all of which play important roles in the ferroptosis pathway. Further validation using real‐time PCR (RT‐PCR) also confirmed the expression differences of some representative genes at the mRNA levels (Figure 4j). Acsl4 is a lipid‐metabolizing enzyme that promotes the production of lipid peroxides. The significant upregulation of Acls4 in PM‐FM/C+US‐treated cells suggested its involvement in promoting ferroptosis. This occurred through the esterification of polyunsaturated fatty acids (PUFAs) to acyl‐coenzyme A, a process further catalyzed by LPCAT3 to generate PUFA‐containing phospholipids, which were prone to peroxidation.^[^ ^27^ ^]^ The reduced transcript level expression of *Gpx4*, a key gene essential for maintaining redox homeostasis and preventing ferroptosis,^[^ ^28^ ^]^ in the PM‐FM/C+US group further supported the inhibition of GPX4 by PM‐FM/C+US treatment, which was consistent with the results validated by Western blot analysis (Figure 3e). Furthermore, the different expression of two genes, *Gclm, Gss*, involved in GSH metabolism and cysteine/methionine metabolism, highlighted the critical role of the cysteine/GSH/GPX4 axis in facilitating ferroptosis induced by PM‐FM/C under US irradiation. TRF/TFRC mediate the cellular uptake of iron to maintain iron homeostasis, with their expression precisely regulated by the cellular iron level.^[^ ^29^ ^]^ Following treatment with PM‐FM/C, intracellular iron was in an overloaded state, which in turn downregulated the expression of *Trf* and *Tfrc*. SLC7A11, a key upstream regulators of ferroptosis, participates in the extracellular uptake of cystine and glutamate release, promotes the synthesis of GSH, and maintains the cellular redox homeostasis to protect the cells from the damage. The exogenous ROS production and GSH depletion caused by US‐triggered PM‐FM/C treatment greatly increased oxidative stress in the cells, which induced SLC7A11 expression in an attempt to re‐establish redox homeostasis.^[^ ^30^ ^]^ Thus, the therapeutic mechanisms of PM‐FM/C mainly involve GPX4 inhibition and lipid peroxides accumulation. At the same time, the cells may engage in feedback regulation of some specific genes, especially upstream genes, to counteract the damage caused by ferroptosis in an effort to maintain homeostasis. RNA‐sequencing results posed a comprehensive insight on the profound impact of US‐enhanced PM‐FM/C treatment on Hepa1‐6 cells, and further verified the involvement of ferroptosis in the therapeutic mechanisms.

### 2.8 In Vivo Antitumor Study in a Subcutaneous Tumor Model

**Figure 5:**
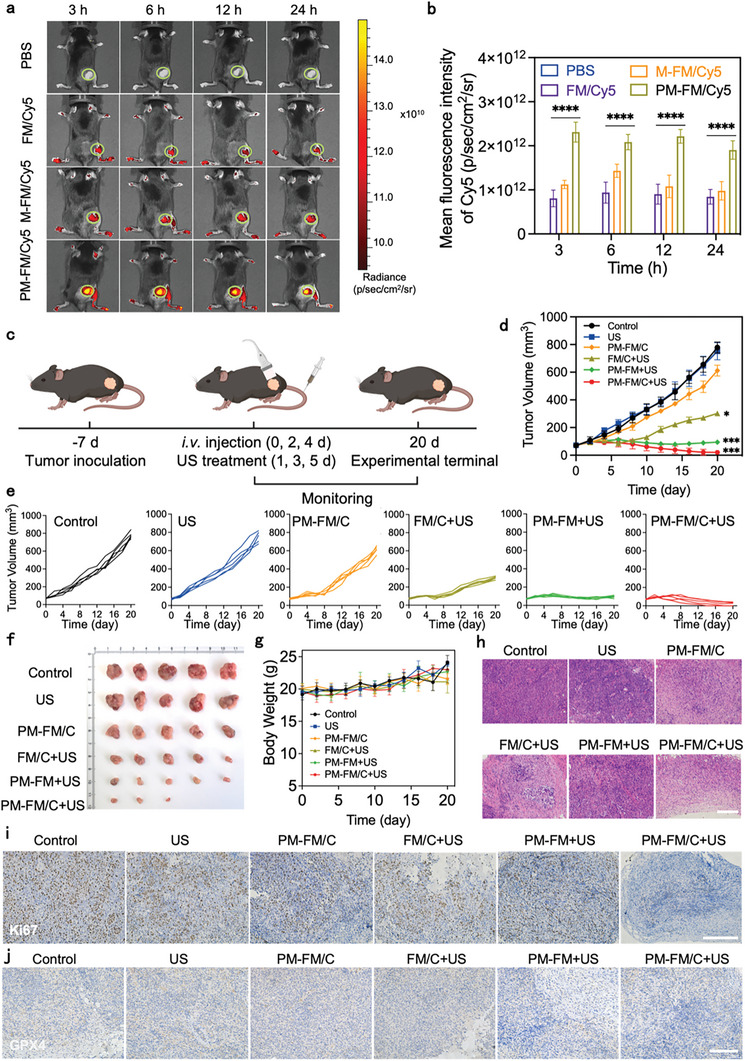
In vivo anti‐tumor effects of PM‐FM/C under US irradiation in a subcutaneous HCC model. a) In vivo fluorescence imaging and b) relative quantification of the fluorescence intensity of Cy5 at different time points after intravenous injection of PBS, FM/Cy5, M‐FM/Cy5 and PM‐FM/Cy5. Data are presented as mean ± SD (*n* = 3 mice). c) Schematic illustration of the therapeutic procedure (*n* = 5). The figure was created with BioRender.com. d) Tumor volume change curves of the Hepa1‐6 tumor‐bearing mice with different treatments. e) Individual tumor growth curves of the Hepa1‐6 tumor‐bearing mice with different treatments. f) Photos of tumor tissues harvested from the mice after different treatments on day 20. g) Body weight of mice in different groups during the experimental periods. H&E staining h), Ki67 staining i), and GPX4 staining j) of tumor tissues harvested from the mice after different treatments. Scale bar: 200 µm. Data are presented as mean ± SD (*n* = 5 mice). ^*^ *p* < 0.05, ^***^ *p* < 0.001, and ^****^ *p* < 0.0001, using two‐way ANOVA. *p* indicates the statistical significance relative to the control group.

Inspired by the excellent therapeutic performance of PM‐FM/C on liver cancer cells by inducing ferroptosis cell death, we further explored the in vivo antitumor effect of PM‐FM/C in the Hepa1‐6 tumor‐bearing mice model. In vivo imaging was first conducted to evaluate the targeting ability of the nanosystem for tumor accumulation. The tumor‐bearing mice were divided into four groups and administrated with PBS, FM/Cy5, M‐FM/Cy5 and PM‐FM/Cy5, respectively, and then imaged at different time points. As depicted in **Figure** **5**a, the tumors exhibited the strongest fluorescence signal at 6 h post‐injection and the signal could be maintained within 24 h. The highest fluorescence signals were observed in the mice treated with PM‐FM/Cy5, indicating effective accumulation via the enhanced permeability and retention effect as well as the enhanced tumor homing effect of the SP94‐modificated macrophage membrane. After 24 h of administration, tumors in the PM‐FM/Cy5‐treated mice exhibited fluorescence signals ≈2.56‐fold and 1.89‐fold higher than those in the M‐FM/Cy5‐treated and FM/Cy5‐treated mice, respectively (Figure 5b).

Then, the antitumor efficacy study was performed according to the procedure shown in Figure 5c. The tumor‐bearing mice were randomly divided into 6 groups (*n* = 5): PBS, US, PM‐FM/C, FM/C+US, PM‐FM+US, PM‐FM/C+US. Nanoparticles were administrated through intravenous injection (*i.v*.) every 2 days for a total of three times, and US irradiation was performed at 24 h post injection. As shown in Figure 5d,e, the tumor growth of the PM‐FM/C+US group was greatly inhibited, with the highest tumor inhibition rate of 97.3% at the end of day 20, which was superior to that of the PM‐FM+US group (87.9%), FM/C+US group (61.1%) and PM‐FM/C group (21.3%). Photo of tumors excised from mice in the different groups further confirmed that PM‐FM/C+US exhibited the most effective anti‐tumor efficacy (Figure 5f). Negligible body weight loss was observed in all groups of mice during the treatment period (Figure 5g), indicating the good biosafety of all treatments.

Hematoxylin and eosin (H&E) histological staining of the tumor tissue sections revealed a higher percentage of necrotic cells in the PM‐FM/C+US group as compared to the other groups (Figure 5h). The reduced expression of Ki67 in the tumor tissue of the PM‐FM/C+US group indicated the effective inhibition of cell proliferation by this treatment (Figure 5i). In addition, the tumor tissue of the PM‐FM/C+US group exhibited the lowest levels of GPX4 expression (Figure 5j), indicating the involvement of ferroptosis during the treatment in vivo. Based on the above results, it can be deduced that the superior antitumor efficacy of PM‐FM/C upon US irradiation was attributed to the enhanced tumor homing due to the dual‐targeting modification, as well as the synergistic effect of CDT/SDT and CO therapy in inducing ferroptosis in tumor cells.

At the end of treatment, the major organs were also harvested for H&E staining, and no noticeable pathological changes were detected (Figure S19, Supporting Information). Moreover, the blood samples of PBS and PM‐FM/C+US groups were collected for biochemical analysis. The values of routine blood parameters and hepatic/renal function indicators showed no significant difference between the PBS‐treated and PM‐FM/C plus US‐treated mice (Figures S20 and S21, Supporting Information). Collectively, the above results demonstrated the excellent therapeutic efficacy and good biocompatibility of US‐triggered PM‐FM/C therapy as a promising therapeutic strategy for HCC treatment in vivo.

### 2.9 In Vivo Antitumor Study in an Orthotopic HCC Mouse Model

**Figure 6:**
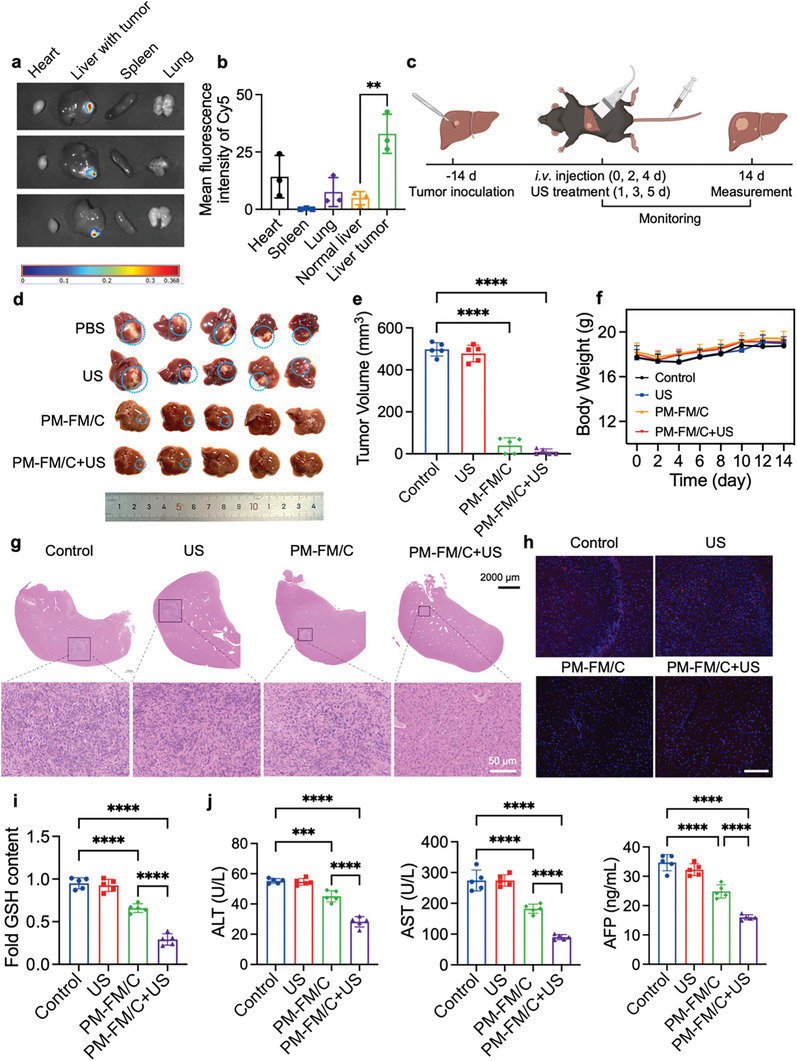
In vivo anti‐tumor effects of PM‐FM/C under US irradiation in an orthotopic HCC model. a) Representative fluorescence imaging and b) quantification of the mean fluorescence intensity of Cy5 in livers with tumor and other major organs harvested from the mice at 12 h after intravenous injection of PM‐FM/Cy5. c) Schematic illustration of the therapeutic procedure (*n* = 5). The Figure was created in BioRender.com. d) Photograph of tumor‐bearing liver tissues harvested from the mice after different treatments. e) Average tumor volume of mice after different treatments. f) Body weight of mice in different groups during the experimental periods. g) Representative H&E staining images of tumor bearing liver tissues after different treatments. h) GPX4 staining of tumor tissues harvested from the mice after different treatments. i) The GSH level in the tumors. j) Biochemical analyses of ALT, AST and AFP of mice after different treatments. Data are presented as mean ± SD (*n* = 5 mice). ^**^ *p* < 0.01, ^***^ *p* < 0.001, ^****^ *p* < 0.0001, using two‐way ANOVA.

Orthotopic tumor models are considered to be more clinically relevant and theoretically predictive than traditional subcutaneous tumor models. Based on the favorable targeting effect observed in the subcutaneous tumor model, we proceeded to evaluate the targeting capability of the nanosystem in orthotopic Hepa1‐6 tumor‐bearing mice. Mice were injected intravenously with PM‐FM/Cy5 and the major organs were harvested for imaging 12 h after injection. As shown in **Figure** **6**a, much stronger fluorescence was observed in the tumor sites compared to the surrounding liver tissues as well as other organs, indicating that the abundant PM‐FM/Cy5 was specifically accumulated in the tumor tissues. The average fluorescence signal of the tumor was significantly higher than that of the normal liver tissue and the tumor/liver signal ratio was calculated to be 6.74 (Figure 6b).

After confirming the excellent targeting ability for orthotopic HCC tumor, we investigated the anti‐tumor effects of PM‐FM/C under US irradiation in the orthotopic model (Figure 6c). The tumor‐bearing mice were sacrificed and the liver tissues were collected at 14 days after the first injection. The digital image and tumor volume of the liver tissues showed that the tumors in PM‐FM/C group were quite small or partially disappeared, and the tumors in PM‐FM/C+US group were almost disappeared (Figure 6d,e), demonstrating that the tumor growth was inhibited by PM‐FM/C to some extent, and the tumor inhibition effect was significantly enhanced by US stimulation. Similar to the subcutaneous model, there was no noticeable body weight loss in all groups of mice (Figure 6f). Besides, the full scanning images of H&E staining of the liver tissue sections clearly displayed that the cancerous area in the liver of PM‐FM/C+US group was greatly reduced compared with PBS and US alone group (Figure 6g). Moreover, GPX4 expression was significantly reduced in PM‐FM/C+US group (Figure 6h). We also detected the GSH level of the tumors from different groups and the results indicated that the GSH level in the tumors of the PM‐FM/C+US group was reduced 3.25‐fold compared with that of the control group (Figure 6i). Based on the results of in vitro experiments, the therapeutic effects might arise from the cascade‐activated catalytic reaction due to the intrinsic H_2_O_2_ in TME for rendering CDT, hypoxia amelioration and CO release, which enhanced the US‐triggered SDT performance to produce synergistic ferroptosis for tumor death.

Finally, H&E staining was also performed on the major organs from the orthotopic model, and no significant pathological changes were observed as well (Figure S22, Supporting Information). Meanwhile, the blood samples were harvested for routine blood and hepatic/renal function evaluation. There was no significant difference in the values of routine blood parameters and renal function between the PBS‐treated and PM‐FM/C plus US‐treated mice (Figures S23 and S24, Supporting Information), indicating the good biocompatibility of the US‐triggered therapy. However, it was noticeable that the level of alanine aminotransferase (ALT), aspartate aminotransferase (AST) and alpha‐fetoprotein (AFP) were significantly increased in the control group because of the tumor burden in the liver, and these indicators recovered to the normal range after PM‐FM/C+US treatment (Figure 6j). These results not only demonstrated the favorable biosafety of the PM‐FM/C+US treatment but also further confirmed its efficacy in reducing tumor burden.

## 3 Conclusion

In summary, a multifunctional nanoplatform based on bimetallic FM and CORM‐401 with SP94 peptide‐inserted macrophage membrane coating was rationally constructed to integrate SDT/CDT and CO gas therapy for HCC treatment. FM was explored as an advanced sonosensitizer with CAT mimicking activity to alleviate tumor hypoxia to achieve excellent SDT performance under US irradiation. Meanwhile, FM possessed POD‐like activity to generate ·OH for CDT. The SP94 peptide‐inserted macrophage membrane modification endowed the nanoplatform with a favorable targeting capability against HCC. The elevated level of intracellular ROS derived from SDT and CDT facilitated the cascade release of CO, along with GSH depletion to induce GPX4, which together led to lipid peroxidation and synergistic ferroptosis in HCC cells. Such a US‐triggered multimodal therapeutic strategy exerted excellent antitumor effect in vitro and significantly inhibited tumor growth in subcutaneous and orthotopic HCC therapy. Overall, this study highlighted a promising strategy to boost synergistic ferroptosis for effective HCC therapy.

## 4 Experimental Section

### Materials

Ferrous sulfate heptahydrate (Na_2_MoO_4_·2H_2_O, purity 99.0%), and sodium molybdate dihydrate (FeSO_4_·7H_2_O, purity 99.0%), 3,3,5,5′‐tetramethylbenzidine (TMB), 9,10‐anthracenediyl‐bis (methylene)dimalonic acid (ABDA), and hemoglobin (Hb) were obtained from Aladdin Biochemical Technology Co., Ltd (Shanghai, China). CORM‐401 (purity 98.27%) purchased from MedChemExpress. DSPE‐PEG_2K_‐Mal (purity 95%) and Sulfo‐Cyanine5 NHS (Cy5 NHS, purity 95%) were provided by Xi'an ruixi Biological Technology Co., Ltd (Xi'an, China). SP94 peptide (purity 97.69%) was obtained from GL Biochem (Shanghai) Ltd (Shanghai, China). RT‐PCR primes of *Acsl4*, *Gclm*, *Slc7a11*, *Slc39a8*, *Gpx4*, and *Gss* were provided by Beijing Tsingke Biotech Co., Ltd (Beijing, China). 5,5′,6,6′‐Tetrachloro‐1,1′,3,3′‐tetraethylbenzimidazolylcarbocyanine iodide (JC‐1) was purchased from Shanghai Maokang Biotechnology Co., Ltd (Shanghai, China). Calcein‐AM/PI cell viability/cytotoxicity assay kit was purchased from Beyotime Biotechnology (Shanghai, China). Cell Counting Kit‐8 (CCK‐8) was provided by Beijing Labgic Technology Co., Ltd (Beijing, China). Liperfluo was purchased from Dojindo Laboratories (Kumamoto, Japan). BODIPY581/591‐C11 was provided by Thermo Fisher Scientific (Massachusetts, USA). 2′,7′‐Dichlorodihydrofluorescein diacetate (DCFH‐DA) was purchased from MedChemExpress (Shanghai, China). Anti‐glutathione peroxidase 4 (GPX4) antibody (ab125066) and Anti‐β actin antibody (ab8227) were provided by Abcam (Cambridge, UK). Rabbit β‐actin mAb (High Dilution) (AC026) was purchased from ABclonal Technology Co., Ltd (Wuhan, China). Anti‐Ki67 Rabbit pAb (GB111141) and anti‐Glutathione Peroxidase 4 Rabbit pAb (GB114327) were obtained from Servicebio (Wuhan, China). TRIeasy Total RNA Extraction Reagent (10606ES60), Hifair III 1st Strand cDNA Synthesis SuperMix for qPCR (gDNA digester plus) (11141ES60), and Hieff Unicon TaqMan multiplex qPCR master mix (11208ES03) were purchased from Yeasen Biotechnology (Shanghai, China). All chemicals were used as received without further purification.

### Characterizations

Dynamic light scattering (DLS) and zeta potential measurements were performed on a Malvern Zetasizer Nano ZS‐90. The N_2_ adsorption–desorption analysis was performed using a Surface Area and Porosimetry Analyzer (ASAP 2460, Micromeritics Instrument Corporatio). The morphology of the FeMoO_4_ was examined using TEM (JEOL JEM1200EX). The chemical composition was determined with XPS (Thermo Scientific K‐Alpha). FTIR spectrum was examined using the fourier transform infrared spectrophotometer (IRTracer‐100, SHIMADZU). UV–vis absorption spectra were obtained with a UV–vis spectrophotometer (UH5700, HITACHI). Oxygen vacancies, singlet oxygen, and electron holes were detected by ESR (A300‐10/12, Bruker). The loading capacity of CORM‐401 was measured using ICP‐MS (ICPMS7800, Agilent). CLSM images were captured using a confocal microscope (LSM710, CARL ZEISS). Flow cytometry assays were performed using a flow cytometer (FACSCalibur, BD).

### Synthesis of FeMoO_4_ Nanoparticles

Typically, Na_2_MoO_4_·2H_2_O (1.2098 g, 5 mmol) and FeSO_4_·7H_2_O (1.3901 mg, 5 mmol) were each dissolved in deionized water (30 mL) and stirred for 20 min. The FeSO_4_ solution was then added dropwise to the Na_2_MoO_4_ solution, followed by stirring for an additional 20 min. The resultant solution was transferred to a Teflon liner (100 mL) and heated at 180 °C for 12 h. The resultant FeMoO_4_ was collected by centrifugation (8000 rpm, 10 min), washed with deionized water and ethanol three times, and dried in an oven (60 °C) for 6 h.

### Macrophage Membrane Isolation

Raw 264.7 cells membrane was collected according to previously reported procedures.^[^ ^31^ ^]^ First, Raw 264.7 cells on the culture dish were washed with PBS. The obtained cells were collected and suspended in the homogenization buffer before being disrupted with a probe ultrasonic disruptor for 10 min at 4 °C (100 W, sonicate for 3 s, and pause for 2 s), followed by centrifugation (20000 *g*, 20 min). The protein content of the cell membrane was measured with the BCA protein assay kit (Omni‐Easy Instant BCA Protein Assay Kit). The prepared cell membrane solutions were stored at −80 °C and used as soon as possible.

### Synthesis of DSPE‐PEG_2K_‐SP94

DSPE‐PEG_2K_‐SP94 was synthesized through a maleimide‐thiol coupling reaction, with a fixed molar ratio of 1:1 between SP94 and DSPE‐PEG_2k_‐Mal. Briefly, predetermined amounts of SP94 and DSPE‐PEG_2k_‐Mal (20 mg) were dissolved in dimethyl sulfoxide (DMSO) (2 mL) and stirred for 24 h at room temperature. Then, the mixture was dialyzed against deionized water overnight to remove DMSO. The resulting aqueous solutions were lyophilized and stored at −20 °C for further use.

### Preparation of SP94‐Modified Macrophage Membrane

For the preparation of the SP94‐modified macrophage membrane, DSPE‐PEG_2k_‐SP94 was dissolved in PBS and applied to the prepared membrane in a 1:2 molar ratio. The mixture was stirred at 4 °C for 30 min before being centrifuged (14000 rpm, 5 min) to remove free DSPE‐PEG_2k_‐SP94. After that, the SP94‐modified membrane was sonicated for 5 min and extruded by an Avanti mini‐extruder. The produced SP94‐modified membrane was stored at −80 °C for future use.

### Preparation of FM/C, M‐FM/C, PM‐FM, PM‐FM/C

FM and CORM‐401 (with a fixed molar ratio of 1:1) were mixed and stirred overnight at room temperature. The mixture was centrifuged at 14000 rpm. Then, the supernatant was discarded, and the pellet was collected to obtain FM/C. The loading efficiency of CORM‐401 in FM/C was determined by ICP‐MS. For the preparation of PM‐FM/C, the SP94‐modified membrane was coated onto the FM/C core by 10 min sonication in a water bath sonicator and then another 5 min sonication with a probe ultrasonic disruptor (100 W, sonicate for 5 s and pause for 5 s). The cell membranes on the as‐prepared PM‐FM/C was validated by sodium dodecyl sulfate‐polyacrylamide gel electrophoresis (SDS‐PAGE).

As controls, FM/C was coated with macrophage membranes without SP94 modification to create single‐targeting nanoparticles (M‐FM/C), while FM was directly coated with SP94‐modified membranes to produce nanoparticles lacking CORM‐401 (PM‐FM). Fluorescence‐labeled nanosystems were prepared following a similar procedure as described above, except that CORM‐401 was replaced with Cy5‐NHS.

### In vitro ^·^OH Generation

To monitor the generation of ·OH, FM and PM‐FM/C (at an equivalent FM concentration of 10 µg mL^−1^) were respectively dispersed in the MES buffer solutions (3 mL, 0.1 mol, pH 5.4) containing TMB (0.25 mmol). H_2_O_2_ was then added to the above solutions to a final concentration of 10 mmol. The absorbance spectra of TMB were measured at different time points using a UV–vis spectrometer.

### O_2_ Release

To evaluate the oxygen release, FM was dispersed in deoxygenated water (10 mL) to prepare FM solutions at various concentrations (1, 2, and 4 mg mL^−1^) at room temperature. H_2_O_2_ was then added to the mixture to give a final concentration of 10 mmol, and the dissolved oxygen was detected using a dissolved oxygen meter. As a control, the dissolved oxygen in deoxygenated water (FM 0 mg mL^−1^) was measured with the addition of H_2_O_2_.

### In Vitro Detection of Singlet Oxygen (^1^O_2_)

FM and PM‐FM/C (at an equivalent FM concentration of 50 µg mL^−1^) were respectively dispersed in deionized water (3 mL) containing ABDA (20 µg mL^−1^). The formulations were treated with US irradiation for 10 min (1 MHz, 50% duty cycle, 1.25 W cm^−2^), and then the UV–vis spectra of ABDA were measured at different time points.

To confirm the production of singlet oxygen in a hypoxic environment, the experimental reagents were purged with nitrogen for 10 min. Then, the respective solutions of FM or PM‐FM/C (at an equivalent FM concentration of 50 µg mL^−1^) were mixed with ABDA, sealed, and subjected to ultrasound irradiation for 10 min (1 MHz, 50% duty cycle, 1.25 W cm^−2^). The UV–vis spectra of ABDA were recorded at various time points.

### Consumption of GSH

The GSH solution (10 mmol, 20 µL) was incubated with a solution (3 mL) containing different concentrations of FM (0, 75, 150, 300, 600, 1200 µg mL^−1^) at 37 °C for 12 h. After that, the DTNB solution (2.5 mg mL^−1^, 100 µL) was added into the mixture and culture at 37 °C for 5 min. The remaining amount of GSH was detected by measuring the absorbance at 412 nm using a microplate reader.

### In Vitro CO Release

The release of CO was measured by the hemoglobin incubation method. CO can bind to hemoglobin, forming carboxyhemoglobin (HbCO), which can be detected by UV–vis spectrometer. The peak at 410 nm of the UV–vis absorbance spectrum was attributed to HbCO while the peak at 430 nm was attributed to Hb. Samples with an equivalent FM concentration of 50 µg mL^−1^ (FM/C, M‐FM/C, and PM‐FM/C) and hemoglobin (4.2 µmol) in PBS solution (3 mL) were bubbled with nitrogen for 10 min, H_2_O_2_ was then added to a final concentration of 10 mmol, and the mixture was immediately enclosed in a UV quartz. Meanwhile, the ultrasound group was treated with US irradiation (1 MHz, 50% duty cycle, 1.25 W cm^−2^). The UV–vis spectra of CO release were respectively detected at 0, 3, 5, 10, 20, 30, 40, 50, 60, 70, 80, 90, and 100 min during the reaction.

### Cells and Animals

The murine macrophage cell line RAW264.7 was purchased from Chinese Academy of Sciences Cells Bank. The murine hepatoma cell line Hepa1‐6 was obtained from American Type Culture Collection. All cells were cultivated in Dulbecco's Modified Eagle Medium (DMEM) medium supplemented with 1% penicillin‐streptomycin (10000 U mL^−1^) and 10% fetal bovine serum (FBS, Cellmax) at 37 °C under a humidified atmosphere supplemented with 5% CO_2_.

Female C57BL/6 mice (6–8 weeks old) were obtained from SiPeiFu Biotechnology Co., Ltd. (Beijing, China). All the protocols were approved by the Committee for Experimental Animals Welfare and Ethics of Institute of Radiation Medicine, Chinese Academy of Medical Sciences (No.IRM/2‐IACUC‐2410‐003). To establish the subcutaneous tumor model, Hepa1‐6 cells (1 × 10^6^ cells / 200 µL) suspended in PBS solution were subcutaneously injected into the back of mice. The orthotopic transplantation tumor models were established using tumor tissues removed from the subcutaneous model. The subcutaneous tumors were excised and cut into pieces, and then similar‐sized tumor pieces were transplanted into the liver of healthy C57BL/6 mice to establish the orthotopic xenograft liver tumor models.

### Cellular Uptake

The cellular internalization was evaluated by flow cytometry and CLSM. For flow cytometry assay, Hepa1‐6 cells were seeded in 6‐well plates (3 × 10^5^ cells per well) and cultivated for 24 h. Fresh DMEM medium containing FM/Cy5, M‐FM/Cy5, and PM‐FM/Cy5 at a Cy5 concentration of 10 µg mL^−1^ was added into each well, and cells were co‐incubated for 4 h. Then, the cells were washed with PBS solution and harvested for flow cytometry analysis. For CLSM detection, Hepa1‐6 cells were incubated in confocal dishes (3×10^5^ cells per dish) overnight. FM/Cy5, M‐FM/Cy5, and PM‐FM/Cy5 (at an equal Cy5 concentration of 10 µg mL^−1^) were then co‐incubated with Hepa1‐6 cells for 2 h, 4 h, and 8 h, respectively. Cells were washed three times with PBS, fixed with paraformaldehyde (4%) solution, stained by 4′,6‐diamidino‐2‐phenylindole (DAPI) (2.5 µg mL^−1^), and detected by CLSM.

### Intracellular ROS Generation

Hepa1‐6 cells were seeded and cultivated in 6‐well plates (3 × 10^5^ cells per well) for 24 h, then co‐incubated with fresh DMEM containing different formulations for another 12 h, including:1) fresh DMEM; 2) US; 3) PM‐FM, 4) PM‐FM/C; 5) PM‐FM+US, and 6) PM‐FM/C+US. US irradiation (1 MHz, 50% duty cycle, 1.25 W cm^−2^) was performed in the groups labelled with US. Then, fresh serum‐free medium containing DCFH‐DA (10 µmol) was added and incubated with the cells for 30 min. The fluorescence intensity was evaluated by CLSM and flow cytometry by detecting the fluorescence intensity of 2,7‐dichlorofluorescein diacetate (DCFH‐DA, Ex/Em = 488/525 nm).

### The Intracellular CO Release

The intracellular CO release was evaluated using a CO probe (COP). The probe was synthesized following a previously reported method.^[^ ^32^ ^]^ As shown in the synthetic route below, CH_3_OH (10 mL) was used to dissolve Rhodamine B (RB) (1 mmol), and then hydrazine hydrate (0.5 mL) was added. The solution was reacted at 75 °C until the color changed from red to uncolored. The mixture was then cooled, concentrated under vacuum, and extracted with distilled water and ethyl acetate. The extract was dried over anhydrous magnesium sulfate, filtered, and vacuum evaporated to yield Compound 1. Subsequently, 1 mmol of Compound 1 was diluted in ethanol (20 mL), and *p*‐dimethylaminobenzaldehyde (0.1688 g) was added gradually. The mixture was agitated and refluxed at 85 °C for 18 h to produce a khaki solid. To get COP, the substance was washed twice with PBS, followed by further refinement using silica column chromatography (petroleum ether: ethyl acetate = 10:1). Hepa1‐6 cells were then seeded in confocal culture dishes (3 × 10^5^ cells per well) for 24 h. The cells were treated with fresh DMEM containing various formulations including 1) fresh DMEM, 2) PM‐FM, 3) PM‐FM/C, and 4) PM‐FM/C+US (1 MHz, 50% duty cycle, 1.25 W cm^−2^) at an equivalent FM concentration of 50 µg mL^−1^ for 12 h. Then, the cells were incubated with fresh PBS solution containing COP probe (40 µg mL^−1^) for 20 min. CLSM (LSM710, CARL ZEISS) was employed to analyze the cells immediately.


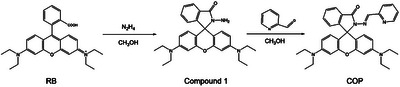


The synthetic route of COP.

### Cytotoxicity Study

Hepa1‐6 cells and 293T cells were seeded in 96‐well plates (5 × 10^3^ cells per well) and cultivated for 24 h. Subsequently, the culture media were substituted with fresh DMEM containing different formulations (PM‐FM, PM‐FM/C) at various concentrations (0, 6.25, 12.5, 25, and 50 µg mL^−1^), and further cultured for 12 h. Then, the cells in the US, PM‐FM+US, and PM‐FM/C+US groups were irradiated with US (1 MHz, 50% duty cycle, 1.25 W cm^−2^) for 5 min. Cell Counting Kit‐8 (CCK‐8) agent was used to detect the cell viability 24 h following US irradiation. The absorbance at 450 nm was detected using a microplate reader to calculate the relative cell viability.

Cell viability was also evaluated using live/dead co‐staining. For this, Hepa1‐6 cells were seeded at 3 × 10^5^ cells per well in 6‐well plates. After incubation at 37 °C for 24 h, the cells were subjected to different treatments: 1) fresh DMEM; 2) US; 3) PM‐FM; 4) PM‐FM/C; 5) PM‐FM+US; 6) PM‐FM/C+US (at an equivalent FM concentration of 50 µg mL^−1^). For the US irradiation groups, cells were treated with US (1 MHz, 50% duty cycle, 1.25 W cm^−2^) after 12 h incubation with different formulations. Following an additional 8 h of incubation, cell viability was assessed using the Calcein/PI cell viability/cytotoxicity assay kit. After 30 min of incubation with the Calcein/PI buffer, the cells were observed under a fluorescent microscope.

### Intracellular GSH Detection

Hepa1‐6 cells were seeded at 3 × 10^5^ cells per well in 6‐well plates. After incubation at 37 °C for 24 h, the Hepa1‐6 cells were given different treatments: 1) fresh DMEM; 2) fresh DMEM+US; 3) PM‐FM; 4) PM‐FM/C; 5) PM‐FM+US; 6) PM‐FM/C+US. For the US irradiation groups, the cells were treated with US (1 MHz, 50% duty cycle, 1.25 W cm^−2^) after 12 h incubation of different formulations. After another 8 h of incubation, the GSH concentration was detected using the Total Glutathione Assay Kit.

### Intracellular GPX4 Expression

The western blotting was performed to assess the intracellular expression of glutathione‐dependent peroxidase 4 (GPX4). Hepa1‐6 cells were seeded in 6 well‐plates at a density of 3 × 10^5^ per well and cultivated for 24 h. Then, the culture medium was replaced, and cells were given different treatments: 1) fresh DMEM, 2) fresh DMEM+US, 3) PM‐FM, 4) PM‐FM/C, 5) PM‐FM+US, and 6) PM‐FM/C+US (with a relative concentration of FM at 50 µg mL^−1^). After 12 h of incubation, the US‐labeled groups were irradiated with US (1 MHz, 50% duty cycle, 1.25 W cm^−2^) for 5 min, while the other groups remained untreated. The cells were then incubated for an additional 12 h. Following treatment, the cells were collected and lysed using RIPA lysis buffer on ice for 30 min. The resulting solutions were centrifuged at 4 °C to obtain the extracted protein solution. The concentration of the extracted protein was determined using a BCA protein assay kit. Subsequently, protein (10 µg) from each group was loaded and subjected to the Simple Western procedure. Signals were detected using a Tanon Gel Imager and quantified with ImageJ software.

### Intracellular LPO Assessment

Hepa1‐6 cells were seeded and cultivated in confocal culture dishes (3 × 10^5^ cells per well) for 24 h before being treated with various formulations (fresh DMEM, fresh DMEM+US, PM‐FM, PM‐FM/C, PM‐FM+US, and PM‐FM/C+US) at an equivalent FM concentration of 50 µg mL^−1^ for 24 h. Then, the corresponding groups were exposed to US irradiation (1 MHz, 50% duty cycle, 1.25 W cm^−2^). After that, the cells were further cultured with BODIPY581/591‐C11 (5 µmol, Ex/Em = 485/520 nm) or Liperfluo (5 µmol, Ex/Em = 524/535 nm) for 20 min and then assessed by CLSM and flow cytometry.

### Mitochondrial Membrane Potential Detection

Hepa1‐6 cells were seeded in confocal culture dishes at a density of 3 × 10^5^ cells per well and cultured for 24 h. Then, the Hepa1‐6 cells were treated with different formulations, including fresh DMEM, fresh DMEM+US, PM‐FM, PM‐FM/C, PM‐FM+US, and PM‐FM/C+US, at an equivalent FM concentration of 50 µg mL^−1^ for 24 h. After 12 h of incubation with various formulations, the US irradiation groups were exposed to US irradiation (1 MHz, 50% duty cycle, 1.25 W cm^−2^). After an additional 4 h of incubation, the cells were stained with JC‐1 probe (10 µg mL^−1^) for 15 min, washed three times with PBS, and subsequently assessed by CLSM.

### In Vivo Imaging

FM/Cy5, M‐FM/Cy5, and PM‐FM/Cy5 (at a Cy5 dose of 2.5 mg kg^−1^) were systemically administered to tumor‐bearing mice via intravenous injection. For the subcutaneous tumor model, the mice were anesthetized using isoflurane, and fluorescence images were acquired at the appointed time points using the IVIS Lumina II imaging system (Caliper). For the orthotopic tumor model, mice were sacrificed 12 h after injection, and the major organs (heart, liver with tumor, spleen, and lung) were harvested for imaging using a Maestro EX imaging system (CRi Maestro).

### Anti‐Tumor Study in Subcutaneous Tumor Model

When the tumor volume reached ≈70 mm^3^, the mice were randomly divided into six groups (*n* = 5 mice per group): 1) PBS; 2) US, 3) PM‐FM/C; 4) FM/C+US; 5) PM‐FM+US; and 6) PM‐FM/C+US. Mice in the PM‐FM/C, FM/C+US, PM‐FM+US, and PM‐FM/C+US groups were administrated with different formulations at an equivalent FM concentration of 15 mg kg^−1^ on day 0, day 2, and day 4. The ultrasound (3 MHz, 50% duty cycle, 1.25 W cm^−2^) was applied to the mice in the US‐labelled groups on day 1, day 3 and day 5. The tumor volumes and body weights of all mice were recorded every two days during the treatment. Tumor volume was calculated by the formula: V = 0.5×L×W^2^ (where L is the longest diameter and W is the shortest diameter perpendicular to L). On day 20, the mice were sacrificed, and major organs and tumors were harvested for further H&E staining and IHC staining. And the blood samples of mice in the PBS group and PM‐FM/C+US group were collected for analyzing various biochemical parameters.

### Evaluation of the Anti‐Tumor Effect in Orthotopic Liver Tumor Model

Orthotopic tumor‐bearing mice were randomly divided into four groups (*n* = 5 mice per group) and given different treatments including 1) PBS, 2) US, 3) PM‐FM/C, and 4) PM‐FM/C+US. Mice in the PM‐FM/C and PM‐FM/C + US groups received PM‐FM/C at a FM dosage of 15 mg kg^−1^ on days 0, 2, and 4. Ultrasound irradiation (3 MHz, 50% duty cycle, 1.25 W cm^−2^) was applied to the upper abdomen of mice in the US group on days 1, 3, and 5. Body weights were recorded every two days throughout the treatment period. At the conclusion of the experiment, mice were sacrificed, and major organs and tumors were collected for subsequent H&E staining, IHC staining, and immunofluorescence (IF) staining. Blood samples from the PBS and PM‐FM/C + US groups were collected for biochemical parameter analysis.

### Statistical Analysis

Statistical analysis in this study was performed using GraphPad Prism 10.0 software. The results were presented as mean ± standard deviation (SD). When comparing the two groups, the Student t‐test was used. When comparing three or more groups, the one‐way ANOVA test was used. The statistical significance was represented as ^*^ *p* < 0.05, ^**^ *p* < 0.01, ^***^ *p* < 0.001, and ^****^ *p* < 0.0001.

## Conflict of Interest

The authors declare no conflict of interest.

## Supporting information



Supporting Information

## Data Availability

The data that support the findings of this study are available from the corresponding author upon reasonable request.
