# Supplementary material for: A Dual‐Targeting Biomimetic Nanoplatform Integrates SDT/CDT/Gas Therapy to Boost Synergistic Ferroptosis for Orthotopic Hepatocellular Carcinoma Therapy
Source: Adv Sci (Weinh). 2025 Jan 9;12(8):2413833. doi: 10.1002/advs.202413833 (PMC11848614; doi:10.1002/advs.202413833)
Supplement: Supplementary file 1 — Supporting Information [file ADVS-12-2413833-s001.docx]

Supporting Information

**A Dual-Targeting Biomimetic Nanoplatform Integrates SDT/CDT/Gas Therapy to Boost Synergistic Ferroptosis for Orthotopic Hepatocellular Carcinoma Therapy**

*Wen Meng, Ting Chen, Xueping Li, Yi Li, Lu Zhang, Yigang Xu, Tianqiang Song*, Ji Qi*, Qingqing Xiong*, Wen Li^*^*


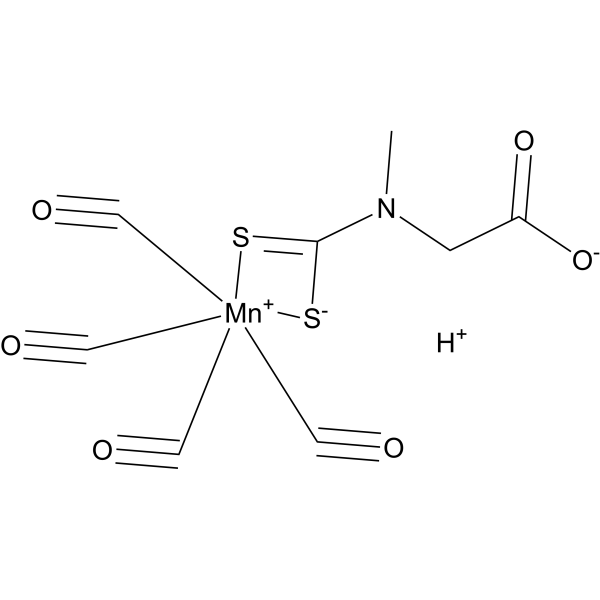


**Figure S1.** The chemical structure of CORM-401.

**Figure S2.** Representative zeta potentials of FM.

**Figure S3.** The FTIR spectrum of FM.


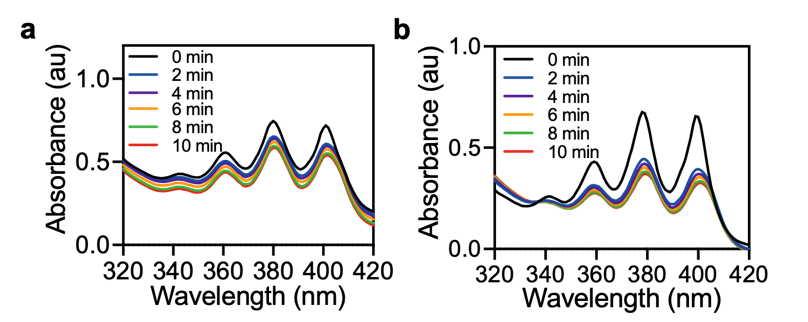


**Figure S4.** The ^1^O_2_ generation ability of FM upon US irradiation under different conditions detected by ABDA (a) Hypoxia at pH 5.4. (b) Hypoxia at pH 5.4 in the presence of H_2_O_2_.


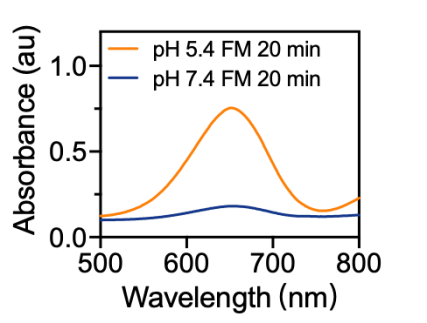


**Figure S5.** The oxidation of TMB by ^•^OH with the treatment of FM (pH = 7.4 and 5.4).


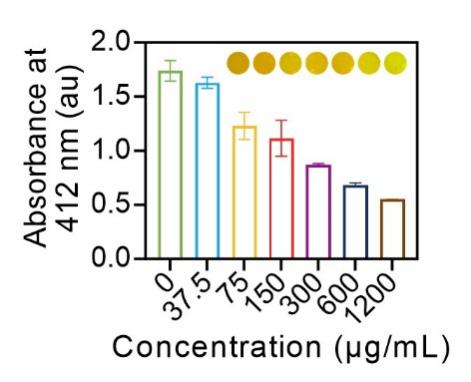


**Figure S6.** GSH depletion by FM of different concentrations (*n* = 3).


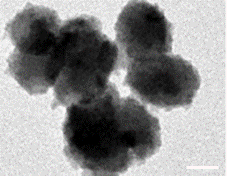


**Figure S7.** TEM image of FM/C. Scale bar: 50 μm.


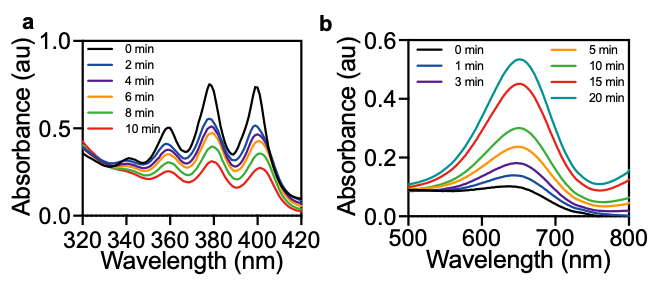


**Figure S8.** (a) The ^1^O_2_ generation ability of PM-FM/C and (b) the oxidation of TMB by ^•^OH with the treatment of PM-FM/C (pH = 5.4).


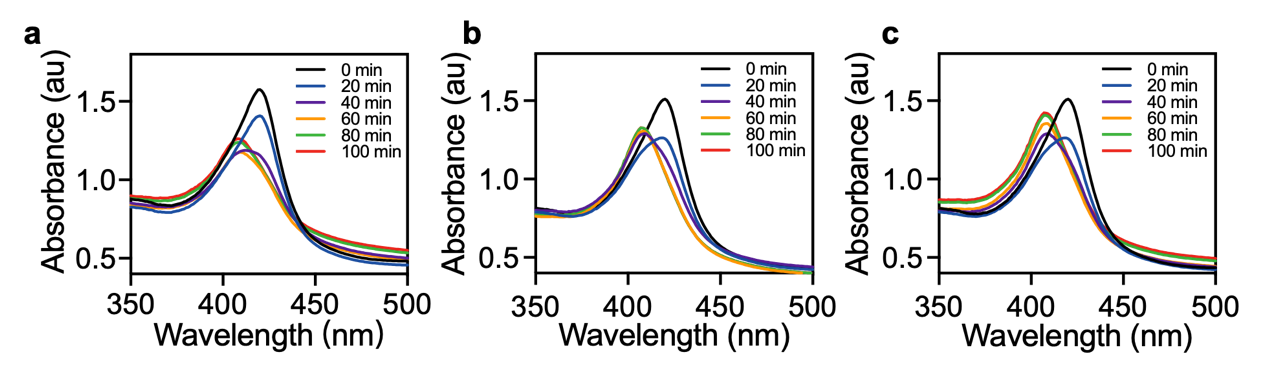


**Figure S9.** The CO release fromb(a) PM-FM/C+US, (b) PM-FM/C+H_2_O_2_, and (c) PM-FM/C+H_2_O_2_+US using Hb as a probe by the UV method.


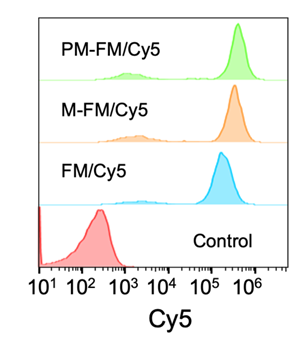


**Figure S10.** Flow cytometry analysis of Cy5 fluorescence detected in Hepa1-6 cells with different treatments as indicated.


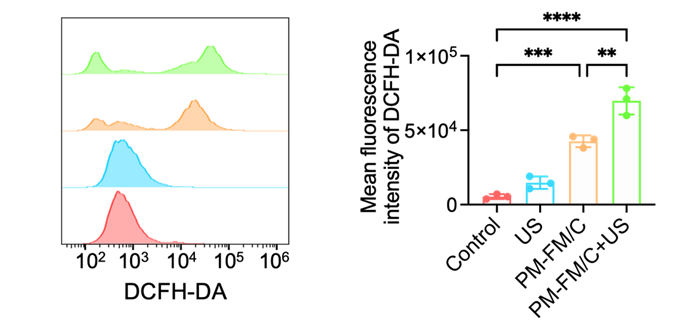


**Figure S11.** Flow cytometry analysis of DCFH-DA fluorescence and representative mean fluorescence intensity detected in Hepa1-6 cells with different treatments as indicated. Data are presented as mean ± SD (*n* = 3). ***p* < 0.01, ****p* < 0.001, and *****p* < 0.0001, and statistical analysis was performed using one-way ANOVA.

**Figure S12.** Cell viabilities of 293T cells treated with various PM-FM or PM-FM/C. Data are presented as mean ± SD (*n* = 3).


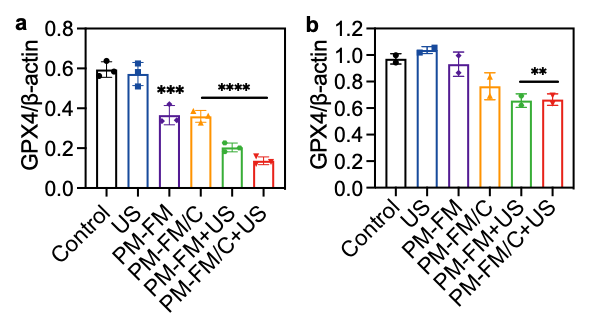


**Figure S13.** Western blot analysis of the expressions of GPX4 in Hepa1-6 cells treated (a) without or with Fer-1 and (b) different treatments as indicated. Data are presented as mean ± SD (*n* = 3 mice). ***p* < 0.01, ****p* < 0.001, and *****p* < 0.0001 using one-way ANOVA. The statistical analysis was calculated compared to the control group.


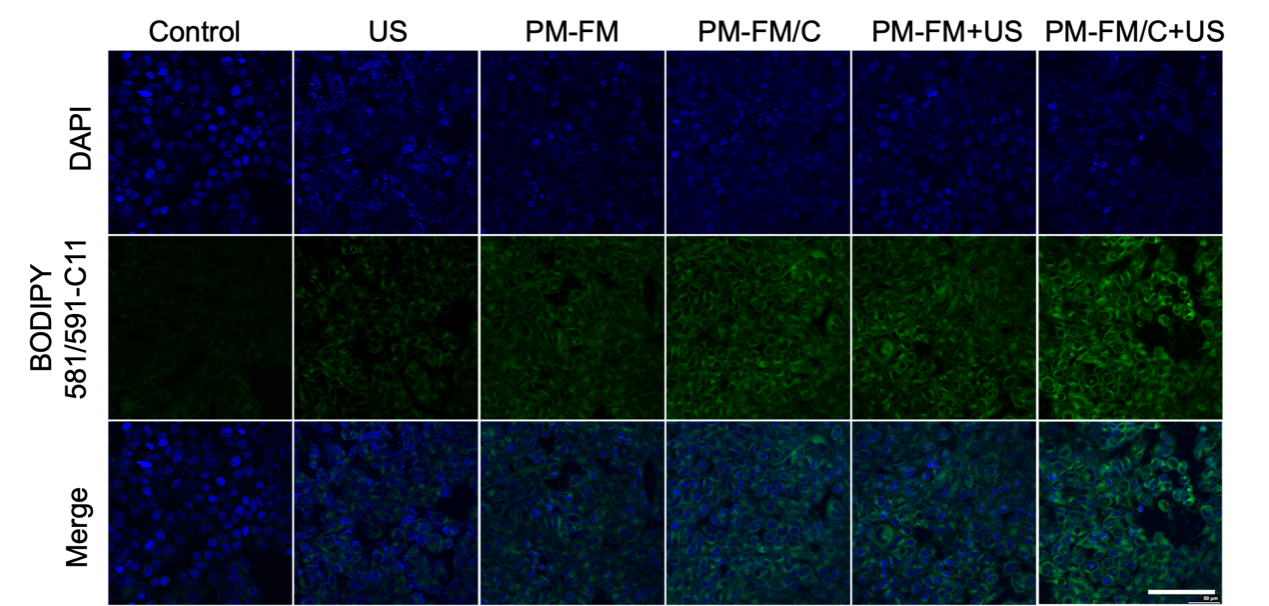


**Figure S14.** Representative CLSM images of lipid peroxidation on Hepa1-6 cells membrane using BODIPY 581/591-C11 as a probe. Scale bar: 100 μm.


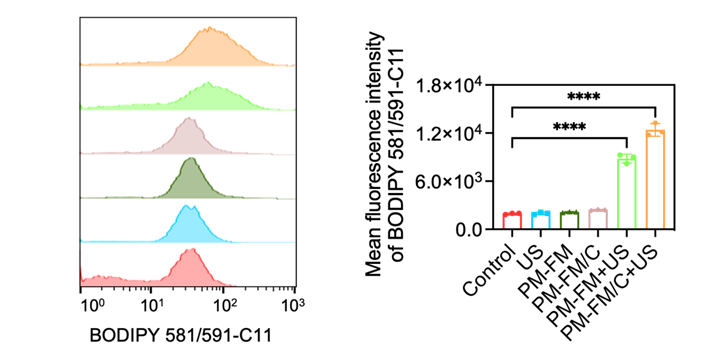


**Figure S15.** Flow cytometry analysis of BODIPY 581/591-C11 in Hep1-6 cells with different treatments. The experiment was repeated three times independently with similar results. Data are presented as mean ± SD (*n* = 3). *****p* < 0.0001, and statistical analysis was performed using one-way ANOVA.

**Figure S16.** Pearson correlation between different samples.


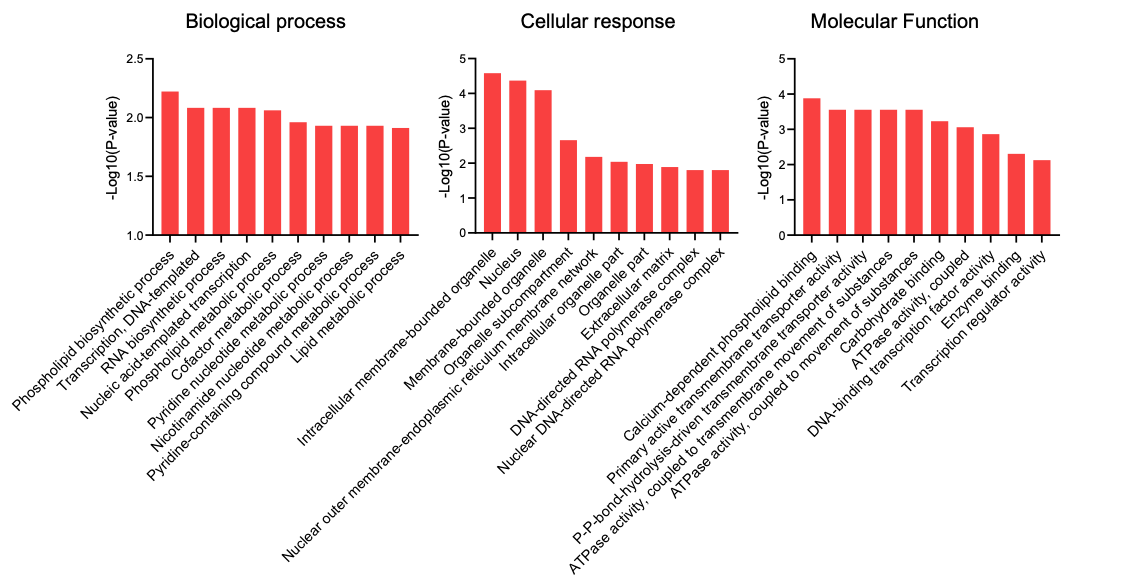


**Figure S17.** GO enrichment analysis between PM-FM/C+US and the control group.


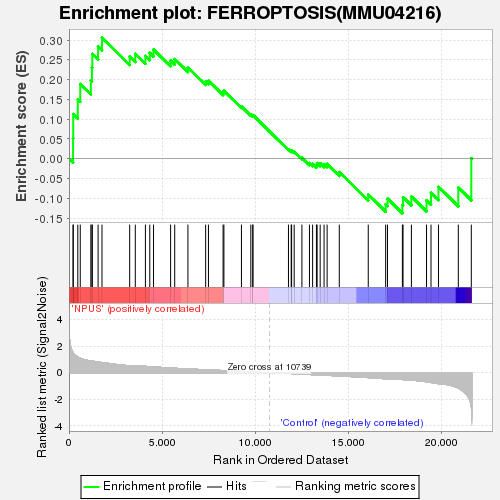


**Figure S18.** GSEA of the DEGs in the ferroptosis signaling pathways.


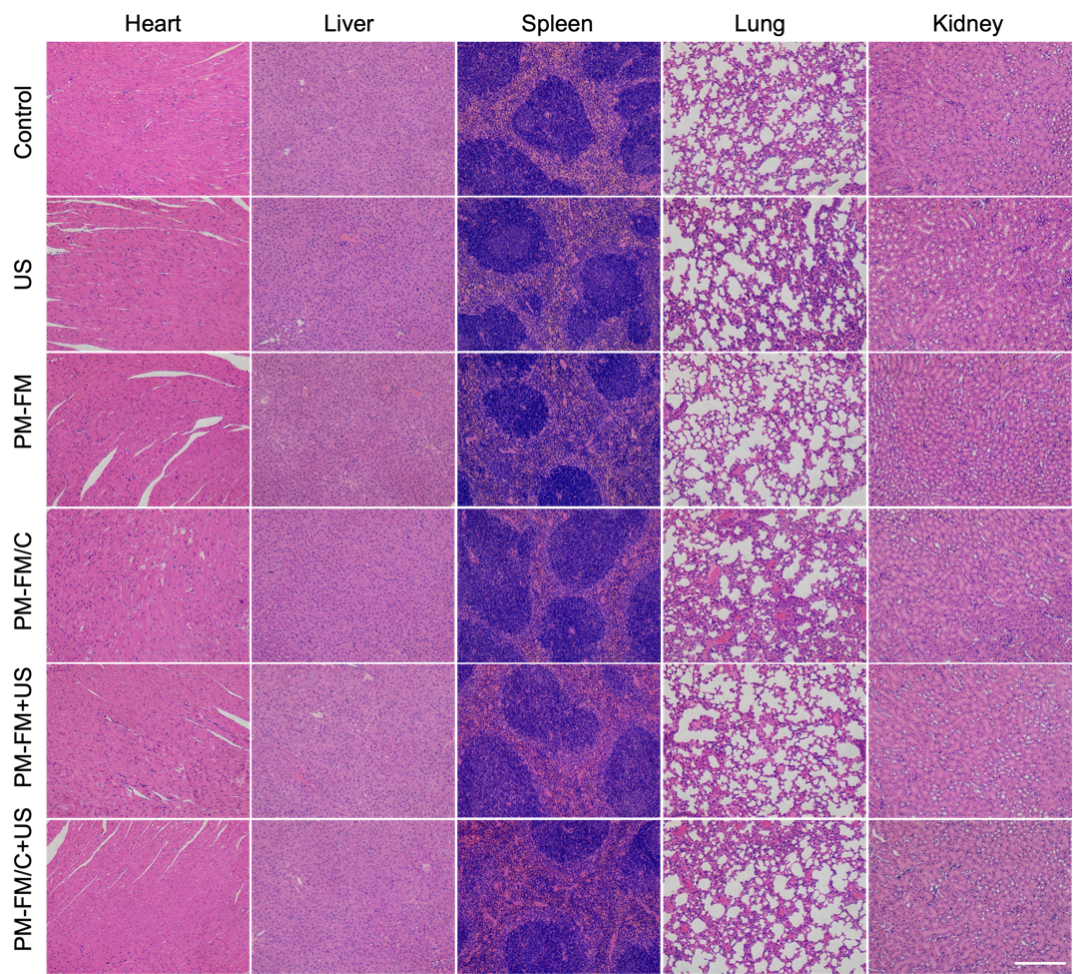


**Figure S19.** Representative H&E staining of heart, liver, spleen, lung and kidney of subcutaneous tumor-bearing mice with different treatments. Scale bar: 100 μm.


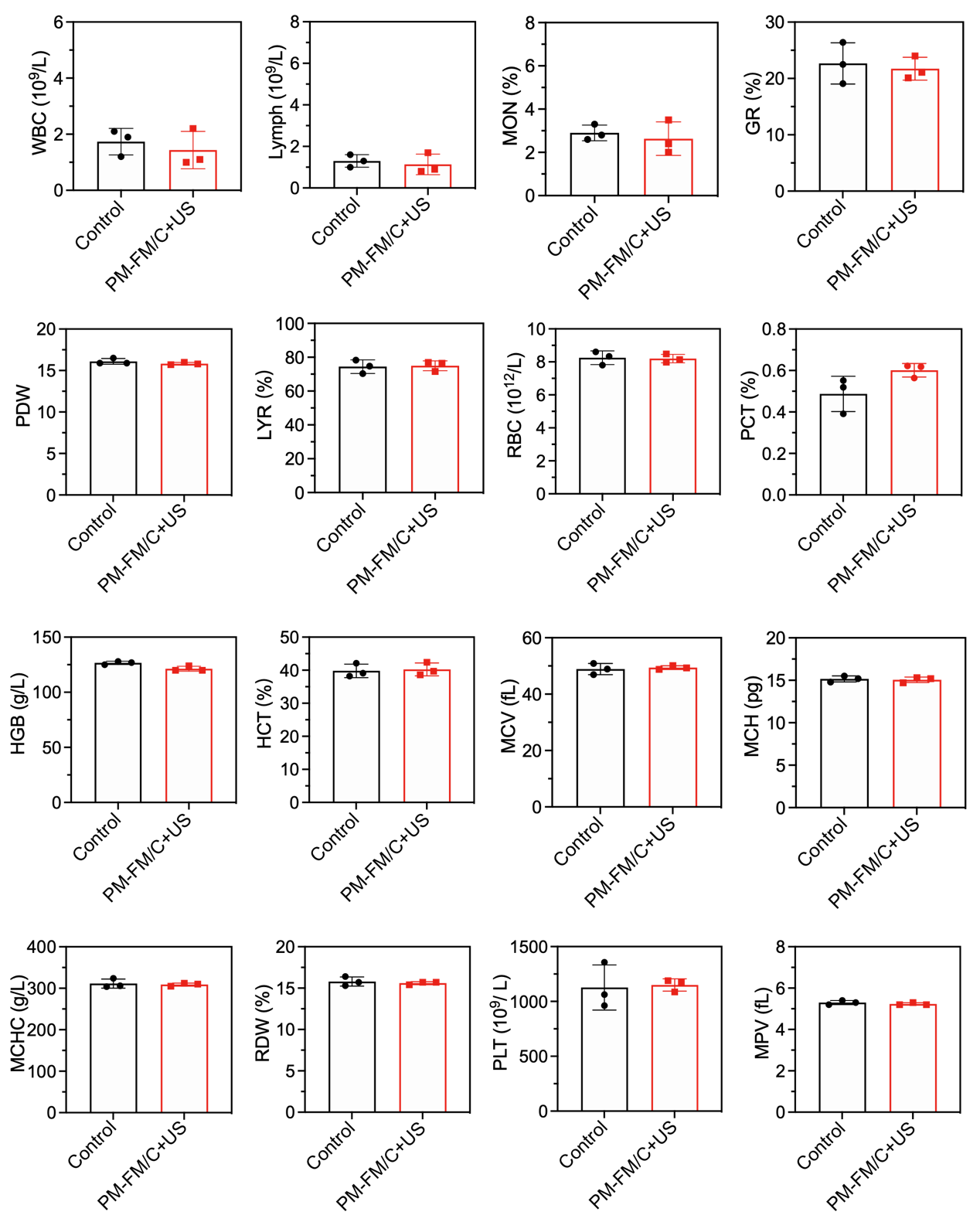


**Figure S20.** Blood routine indexes (white blood cell count (WBC), lymphocyte (Lymph), monocyte (MON), granulocyte (GR), platelet distribution width (PDW), Lymphocyte (LYR), red blood cell count (RBC), plateletcrit (PCT), hemoglobin (HGB), hematocrit (HCT), mean corpuscular volume (MCV), mean corpuscular hemoglobin (MCH), mean corpuscular concentration (MCHC), red blood cell distribution width (RDW), platelets (PLT), and mean platelet volume (MPV)) of the subcutaneous tumor-bearing mice with the treatment of PBS or PM-FM/C+US. Data are presented as mean ± SD (*n =* 3 mice).


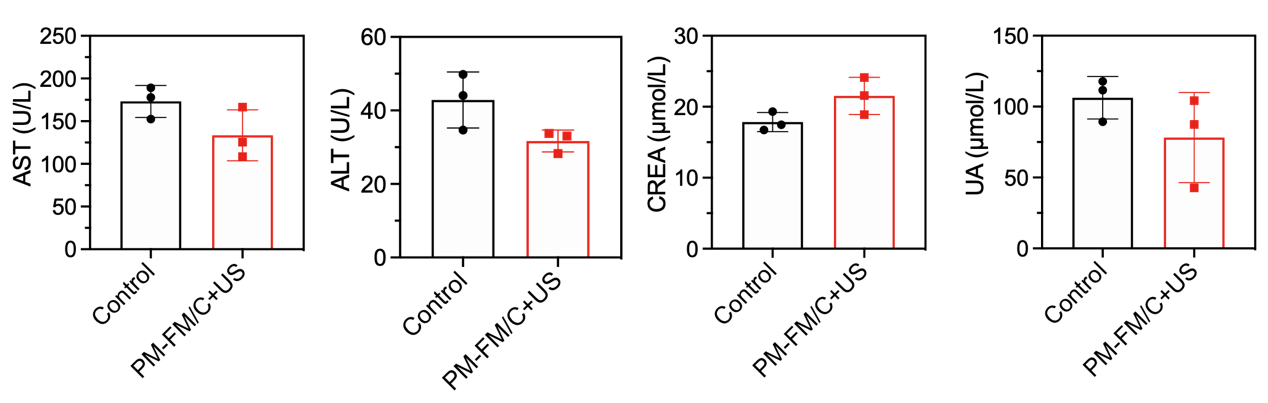


**Figure S21.** Blood test parameters included liver and renal function indexes (aspartate transaminase (AST), Alanine transaminase (ALT), creatinine (CREA), and uric acid (UA)) of the subcutaneous tumor-bearing mice with the treatment of PBS or PM-FM/C+US. Data are presented as mean ± SD (*n* = 3 mice).


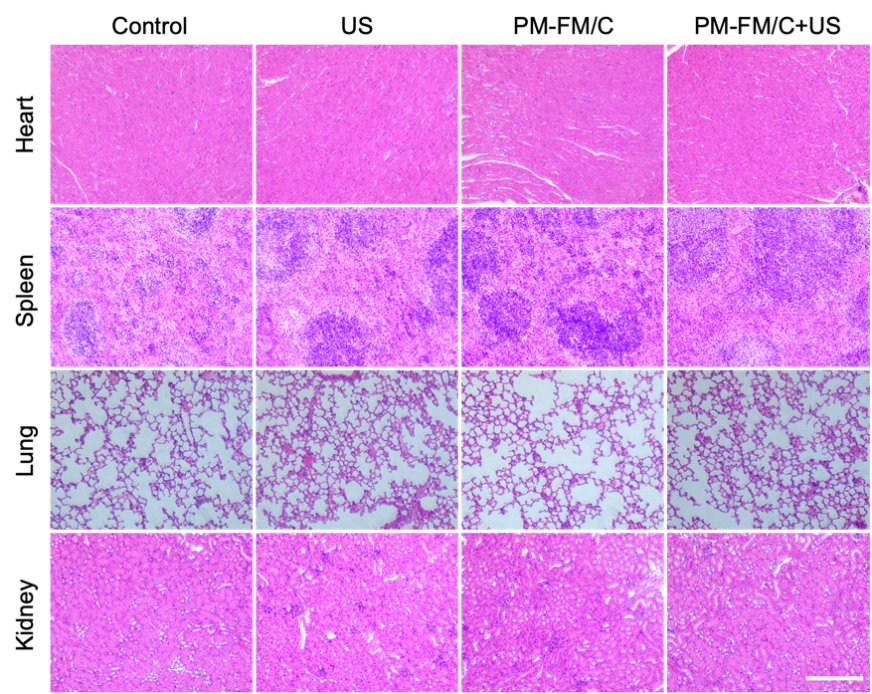


**Figure S22.** Representative H&E staining of heart, spleen, lung and kidney of orthotopic tumor-bearing mice with different treatments. Scale bar: 100 μm.


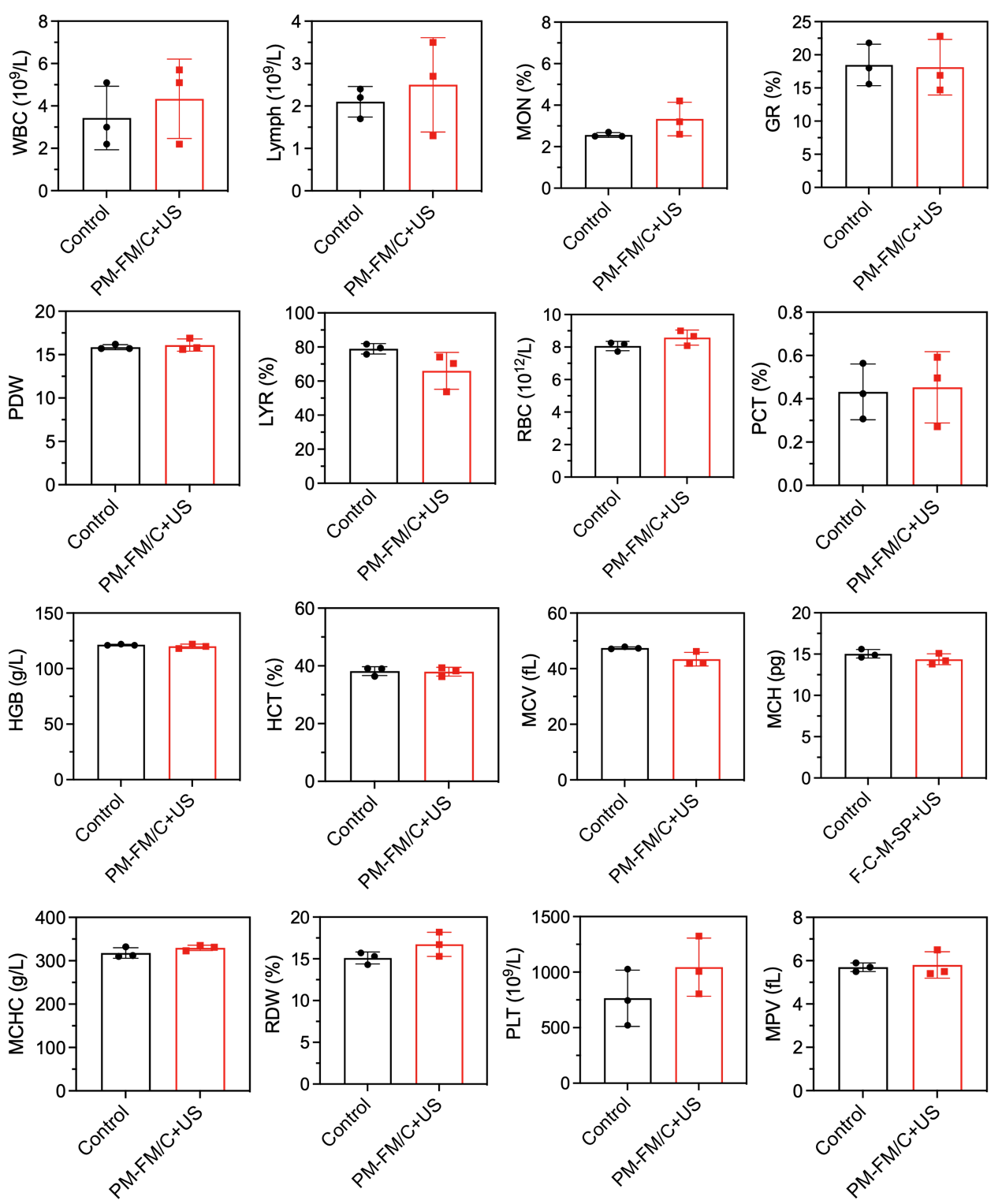


**Figure S23.** Blood routine indexes (white blood cell count (WBC), lymphocyte (Lymph), monocyte (MON), granulocyte (GR), platelet distribution width (PDW), Lymphocyte (LYR), red blood cell count (RBC), plateletcrit (PCT), hemoglobin (HGB), hematocrit (HCT), mean corpuscular volume (MCV), mean corpuscular hemoglobin (MCH), mean corpuscular concentration (MCHC), red blood cell distribution width (RDW), platelets (PLT), and mean platelet volume (MPV)) of the orthotopic tumor-bearing mice with the treatment of PBS or PM-FM/C+US. Data are presented as mean ± SD (*n* = 3 mice).


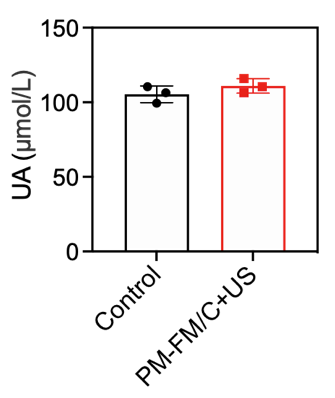

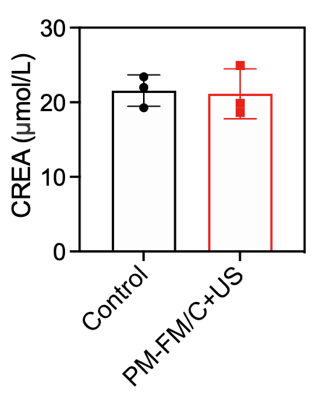


**Figure S24.** The renal function indexes (uric acid (UA) and creatinine (CREA)) of orthotopic tumor-bearing mice with different treatments (*n* = 3 mice).
